# Ectopic USP15 expression inhibits HIV-1 transcription involving changes in YY1 deubiquitination and stability

**DOI:** 10.3389/fcimb.2024.1371655

**Published:** 2024-11-18

**Authors:** Sahar Rezaei, Khalid A. Timani, Ying Liu, Johnny J. He

**Affiliations:** ^1^ Department of Microbiology and Immunology, Rosalind Franklin University, Chicago Medical School, North Chicago, IL, United States; ^2^ Center for Cancer Cell Biology, Immunology and Infection, Rosalind Franklin University, North Chicago, IL, United States; ^3^ School of Graduate and Postdoctoral Studies, Rosalind Franklin University, North Chicago, IL, United States

**Keywords:** USP15, ubiquitin variant inhibitors, deubiquitination, HIV-1 transcription, transcription factors, YY1 protein stability

## Abstract

**Introduction:**

Protein homeostasis is maintained by the opposing action of ubiquitin ligase and deubiquitinase, two important components of the ubiquitin-proteasome pathway, and contributes to both normal physiological and pathophysiological processes. The current study aims to delineate the roles of ubiquitin-specific protease 15 (USP15), a member of the largest deubiquitinase family, in HIV-1 gene expression and replication.

**Methods:**

We took advantage of highly selective and specific ubiquitin variants (UbV), which were recently designed and developed for USP15, and ascertained the inhibitory effects of USP15 on HIV-1 gene expression and production by transfection and Western blotting. We also used real-time RT-PCR, transcription factor profiling, subcellular fractionation, immunoprecipitation followed by Western blotting to determine the transcription factors involved and the underlying molecular mechanisms.

**Results:**

We first confirmed the specificity of USP15-mediated HIV-1 gene expression and virus production. We then showed that the inhibition of HIV-1 production by USP15 occurred at the transcription level, associated with an increased protein level of YY1, a known HIV-1 transcription repressor. Moreover, we demonstrated that USP15 regulated YY1 deubiquitination and stability. Lastly, we demonstrated that YY1 siRNA knockdown significantly diminished the inhibition of USP15 on HIV-1 gene expression and virus production.

**Conclusion:**

These findings together demonstrate that stabilization of YY1 protein by USP15 deubiquitinating activity contributes to USP15-mediated inhibition of HIV-1 transcription and may help the development of USP15-specific UbV inhibitors as an anti-HIV strategy.

## Graphical Abstract

YY1 directly binds to the putative DNA-binding sites (-17 to +27 nt. and -140 to -120 nt.) within the HIV-1 LTR promoter or interacts with other transcription factors that have DNA-binding sites within the HIV-1 LTR promoter. YY1 homeostasis is maintained by the balance of the activities of deubiquitinase USP15 and E3 ubiquitin ligase. Ectopic USP15 expression deubiquitinates and stabilizes endogenous YY1 and thus inhibits HIV-1 transcription, while USP15-specific UbV inhibitors prevent endogenous USP15 from deubiquitinating YY1, thus promoting YY1 degradation and relieving USP15-induced inhibition of HIV-1 transcription.

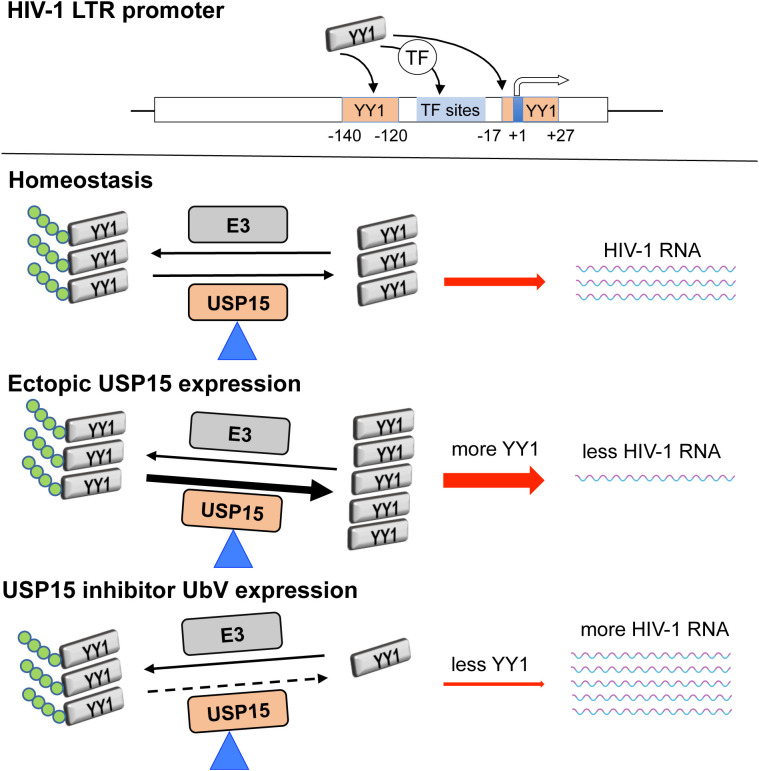

## 1 Introduction

The ubiquitin-proteasome pathway starts with a sequential reaction by three ubiquitin enzymes: ubiquitin-activating enzyme E1, ubiquitin-conjugating enzyme E2, and ubiquitin ligase E3 to add ubiquitin molecules to protein substrates, which is called ubiquitination, and ends with degradation of ubiquitinated proteins in the 26S proteasome complex (McClellan et al., 2019; Damgaard, 2021). Ubiquitination is a reversible process, and the ubiquitin molecules on the ubiquitinated proteins can be removed by ubiquitin-specific deubiquitinating enzymes called deubiquitinases (DUB) (Swatek and Komander, 2016; Mevissen and Komander, 2017; Jacomin et al., 2018). The balance of action between ubiquitin ligase E3 and deubiquitinating enzymes DUB determines the extent of ubiquitination of targeted proteins and, therefore, the fate of the proteins (Li et al., 2018; Woo and Kwon, 2019). Thus, this ubiquitin-proteasome pathway is pivotal for maintaining the homeostasis of cellular proteins and the normal biological function of these proteins, and conceivably, its dysregulation may lead to pathophysiological changes and cause various diseases (Alonso et al., 2011; Brehm and Krüger, 2015; Sun et al., 2020; Liu et al., 2021; El-Saafin et al., 2022; Ye et al., 2023; Zheng et al., 2023). The development of DUB inhibitors as a therapeutic strategy is being actively pursued (Harrigan et al., 2018).

The ubiquitin-proteasome pathway is involved in almost every stage of the human immunodeficiency virus type 1 (HIV-1) life cycle, from the entry of the virus to the release of virions with the interplay and reciprocal manipulation between the cellular proteasome system and HIV-1 proteins (Seissler et al., 2017; Lata et al., 2018; Rojas and Park, 2019). It has also been implicated in HIV-1 latency, as the inhibition or downregulation of proteasomal subunits could reactivate HIV-1 in latent cell line models as well as latent primary CD4+ T cells obtained from virally suppressed individuals under antiretroviral therapy (Li et al., 2019). The ubiquitin-specific proteases (USP) are the largest and well-studied subclass of the DUB family in humans and have more than 50 members (Komander et al., 2009; Cornelissen et al., 2014; Hermanns et al., 2018; Clague et al., 2019). Notably, more studies have shown the regulatory effects of USP members as part of the proteasome machinery on viral protein stability and viral replication, including HIV-1. USP15 stabilizes hepatitis B virus X protein, increases its transcription activity (Su et al., 2017), and stabilizes human papillomavirus type 16 E6 protein through direct interaction (Vos et al., 2009; Yaginuma et al., 2018). USP15 regulates hepatitis C virus RNA translation and lipid metabolism and enhances viral replication (Kusakabe et al., 2019). USP7 and USP47 have modulatory or enhancement effects on HIV-1 (Ali et al., 2017; Setz et al., 2017), while USP3, USP8, USP21, and USP49 (Pan et al., 2019; Gao et al., 2021a, 2021; Zhao et al., 2022) inhibit HIV-1 replication, gene expression or infectivity.

In a collaborative study, we have shown that USP15 selectively targets HIV-1 regulatory protein Nef and structural protein Gag for degradation and inhibits HIV replication solely based on ectopic USP15 expression (Pyeon et al., 2016). USP15 has also been identified as one of the host cell factors taking part in the maintenance of HIV-1 latency through a genetic screening (Röling et al., 2021). Because of high structural and sequence similarity between USP15 and two other USP members, USP4 and USP11 and potential functional overlaps among them (de Jong et al., 2006; Komander et al., 2009; Chou et al., 2017; Teyra et al., 2019), in the current study we took advantage of recently developed ubiquitin variants (UbV) (Teyra et al., 2019), which are highly selective and specific for USP15, to assess the specificity of the inhibitory effects of USP15 on HIV-1 gene expression and virus production and further delineate the underlying molecular mechanisms. Using the USP15 UbV inhibitors, we first confirmed the specificity of USP15-mediated HIV-1 gene expression and virus production. We then showed that the inhibition of HIV-1 production by USP15 occurred at the transcription level, associated with an increased protein level of YY1, a known HIV-1 transcription repressor. Lastly, we demonstrated that USP15 regulated YY1 deubiquitination and stability. These findings provided new mechanistic insights into the roles of USP15 in HIV-1 gene expression and replication and may help develop USP15-targeted anti-HIV therapeutics.

## 2 Materials and methods

### 2.1 Cell culture, plasmids, and transfection

Human embryonic kidney cell line 293T was purchased from the American Type Culture Collection (ATCC, Manassas, VA). Human CD4^+^ T lymphocyte cell line Jurkat (E6-1) (#ARP-177) (Weiss et al., 1984), and HIV-1 infectious molecular clones pNL4-3 (ARP-114) (Adachi et al., 1986), p89.6 (ARP-3552) (Collman et al., 1992), and pYU-2 (ARP-1350) (Li et al., 1991, 1992) were obtained through the NIH HIV Reagent Program, currently integrated into the BEI Resources, NIAID, NIH. pcDNA3 was purchased from Clontech (Mountain View, CA). pUSP15-Myc was generated and described previously (Timani et al., 2014, 2022). pHA-ubiquitin (pHA-Ub) was from Dr. Mark Hannink of the University of Missouri, Columbia, MO (Zhang et al., 2004, 2005). pFlag-UbV.15.1d (pFlag-UbV15.1) and pFlag-diUbV.15.1/D (pFlag-UbV15.1/D) were from Dr. Sachdev S. Sidhu of University of Toronto, Ontario, Canada (Teyra et al., 2019). YY1 siRNA (#sc-36863) and control siRNA (#sc-37007) were both from Santa Cruz Biotechnology (Dallas, TX); USP15 On-Target plus siRNA #2/#4 (#J-006066-06-002/J006066-08-0002) and On-Target control siRNA (D-001810-10-05) were from Horizon Discovery, previously known as Dharmacon (Lafayette, CO). 293T and Jurkat were cultured in Dulbecco’s Modified Eagle’s Medium (DMEM) (Corning, Manassas, VA) and Roswell Park Memorial Institute (RPMI 1640) (Corning), respectively. Both cells were cultured in a 5% CO_2_ and 37°C incubator, and both media were supplemented with 10% fetal bovine serum (R&D Systems, Minneapolis, MN), and 1% *penicillin*/*streptomycin* (Corning). 293T were transfected using the calcium phosphate precipitation method (Chen and Okayama, 1987) or Lipofectamine™ 3000 according to the manufacturer’s instructions (#L3000015, Invitrogen, Carlsbad, CA). Jurkat were transfected using a human T cell Nucleofector™ kit (#VPA-1002, Lonza, Walkersville, MD) and an Amaxa Biosystems Nucleofector II Electroporator (Lonza) according to the manufacturer’s instructions. Carfilzomib was from Cayman Chemical (#17554, Ann Arbor, MI).

### 2.2 Cell lysate preparation, Western blotting, and immunoprecipitation

For whole cell lysates, cells were washed twice with ice-cold phosphate-buffered saline (PBS), suspended in a lysis buffer [10 mM Tris.HCl, pH 8.0, 140 mM NaCl, 1% Triton X-100, 0.1% sodium deoxycholate, 0.1% SDS, 1 mM EDTA, 0.5 mM EGTA, 1 mM PMSF, and 1X Pierce™ protease and phosphatase inhibitor cocktail (#A32959, ThermoFisher Scientific)], and incubated on ice for 20 min. The cell lysates were spun at 12,000 *x g* for 15 min, and the clear upper phase was collected and saved as the whole cell lysates. The protein concentration of lysates was determined using a DC protein assay kit (Bio-Rad, Hercules, CA) and a microplate reader (iMark, Bio-Rad). The proteins in the lysates were analyzed by sodium dodecyl sulfate-polyacrylamide gel electrophoresis (SDS-PAGE), followed by transferring onto a polyvinylidene difluoride (PVDF) membrane, blocking with 5% non-fat dry milk at room temperature (RT for 1 hr), and probing with an appropriate primary antibody (4°C, overnight) and an appropriate secondary antibody (RT for 2 hr). The membranes were then visualized using Pierce™ enhanced chemiluminescent substrates (#32106, ThermoFisher Scientific) and imaged by a ChemiDoc MP imaging system (Bio-Rad). For Immunoprecipitation (IP), whole cell lysates were prepared as above except using a different lysis buffer [50 mM Tris.HCl, pH 8.0, 0.5% NP-40, 280 mM NaCl, 10% glycerol, 0.2 mM EDTA, 2 mM EGTA, 2 mM PMSF, and 1X Pierce™ protease and phosphatase inhibitor cocktail (#A32959, ThermoFisher Scientific)] (Timani et al., 2022). Cleared lysates (700 µg protein) were incubated with a primary antibody (0.7 µg) on a rotator at 4°C overnight, added 30 µl of a mixture of protein A-Agarose (#sc-2001, Santa Cruz Biotechnology) and protein G PLUS-Agarose (#sc-2002, Santa Cruz Biotechnology) (1:1 ratio), and continued to incubate for 1.5 hr. The agarose beads were recovered by centrifugation at 13,500 *x g* for 1 min, washed three times with the lysis buffer, suspended in 20 µl loading buffer, heated 95-100°C for 5 min, and analyzed by Western blotting. Antibodies were: mouse monoclonal antibody GAPDH (6C5) (#sc-32233, Santa Cruz Biotechnology), mouse monoclonal antibody β-actin (#A1978, Sigma-Aldrich, St. Louis, MO), anti-HIV-1 p24 hybridoma (183-H12-5C) (#ARP-1513, NIH HIV Reagent Program) (Chesebro et al., 1992), anti-USP15 (2D5) (#sc-100629, Santa Cruz Biotechnology), anti-Flag^®^ M2 monoclonal antibody (#F3165, Sigma-Aldrich), Histone H3 polyclonal antibody (#PA5-16183, Invitrogen), HA-Tag antibody (F-7) (#sc-7392, Santa Cruz Biotechnology), mouse mAb IgG1 isotype control G3A1 (#5415, Cell Signaling Technology, Danvers, MA), rabbit mAb IgG isotype control DA1E (#3900, Cell Signaling Technology), YY1 rabbit mAb D5D9Z (#46395, Cell Signaling Technology), SRF rabbit mAb D71A9 (#5147, Cell Signaling Technology), goat anti-rabbit HRP-conjugated IgG, (#7074, Cell Signaling Technology) and horse anti-mouse HRP-conjugated IgG (#7076, Cell Signaling Technology).

### 2.3 Reverse transcriptase (RTase) activity assay

The RTase activity assay was performed as previously described (Rezaei et al., 2024). Briefly, one milliliter (ml) culture medium of the cells containing HIV-1 viruses was collected 48 hr post-transfection and centrifuged at 500 *x g* for 5 min to remove cell debris. The clear supernatants were then centrifuged at 21,300 *x g* at 4°C for 90 min, and the virus pellets were suspended in 10 µl dissociation buffer (50 mM Tris.HCl, pH 7.5, 20% glycerol, 0.25% Triton X-100, 0.25 M KCl, and 1 mM Dithiothreitol), subjected to three cycles of quick freeze-thaw, and added the RT assay reaction mixture (40 µl) containing 5 µl poly(A) x (dT)_15_ (# 10108677001, Roche Diagnostics, Indianapolis, IN), 34 µl RT assay buffer (74 mM Tris.HCl pH 7.5, 0.074% Triton X-100, 11 mM MgCl_2_, and 7 mM Dithiothreitol), plus 1 µl deoxythymidine 5’-triphosphate tetrasodium salt-[Methyl-^3^H] (# NET221X005MC, PerkinElmer, Boston, MA). The mixtures were incubated at 37°C for 1 hr and spotted on nitrocellulose membranes (Bio-Rad, Hercules, CA). The membranes were washed three times with 2X saline-sodium citrate buffer (0.3 M sodium chloride, 30 mM sodium citrate, pH 7.0), rinsed twice in 100% ethanol, and air-dried. The ^3^H radioactivity of the membranes was counted in the MicroScint-PS counting fluid (#6013631, PerkinElmer) using a MicroBeta^2^ counter (2450 Microplate Counter, PerkinElmer). The RTase activity was expressed as counts per minute (CPM).

### 2.4 RNA isolation, reverse transcription, and real-time PCR

Total RNA was isolated using TRIzol™ (#15596018, Invitrogen) according to the manufacturer’s instructions and further extracted with acid phenol:chloroform:IAA (125:24:1) (#AM9722, Invitrogen) to prevent residual DNA from being PCR amplified. An iScript™ Reverse Transcription Supermix kit (#1708890, Bio-Rad) was used to synthesize cDNA. qRT-PCR was performed using SYBR Green master mix (#1725270, Bio-Rad) (Rezaei et al., 2024), Applied Biosystems™ 7500 Real-Time PCR System, and primers 5’-AGT GGG GGG ACA TCA AGC AGC CAT GCA AAT-3’ and 5’-TTT GGT CCT TGT CTT ATG TCC AGA ATG C-3’ for HIV unspliced RNA (gag-pol) (ten Haaft et al., 1995; Naitou et al., 1997), 5’-CAG ATG CTG CAT ATA AGC AGC TG-3’ and 5’-TTT TTT TTT TTT TTT TTT TTT TTT TTG AAG-3’ for the total HIV RNA (unspliced and spliced RNA) (Shan et al., 2013); 5′-ACG GCT TCG AGG ATC AGA TTC-3′ and 5′-TGA CCA GCG TTT GTT CAA TGT-3′ for YY1 mRNA (Xu et al., 2020), and 5’-GAA ACT GTG GCG TGA TGG C-3’ and 5’- CCA GTG AGC TTC CCG TTC AG-3’ for GAPDH mRNA, which was included as an internal control for normalization. The quantitation-comparative C_T_ (ΔΔC_T_) program on ABI 7500 was used to calculate the relative target quantity (RQ) compared to the reference sample, normalized to GAPDH, and expressed/graphed as RQ Min and RQ Max as computed using the standard deviation of replicates.

### 2.5 Nuclear/cytoplasmic lysate preparation and transcription factor (TF) activation profiling array

The transfected cells in a culture plate were washed with ice-cold PBS and suspended in a 1:1 ratio mixture of ice-cold PBS and CE buffer [20 mM HEPES, pH 7.8, 0.15% NP-40, 120 mM KCl, 20% glycerol, 2 mM EDTA, 2 mM DTT, and 1X Pierce™ protease and phosphatase inhibitor cocktail (#A32959, ThermoFisher Scientific)]. The cell suspension was gently mixed on an orbital shaker at 4°C for 10 min with intermittent pipetting, transferred to a 1.5 ml microtube, and incubated on ice for 5 min. The samples were centrifuged at 2,350 *x g* at 4°C for 5 min, and the supernatants were collected and saved as cytoplasmic lysates. The pellets were washed twice with ice-cold PBS and suspended in a nuclear extraction buffer [20 mM Tris.HCl, pH 8, 400 mM NaCl, 1% Triton X-100, 20% glycerol, 1 mM DTT, and 1X Pierce™ protease and phosphatase inhibitor cocktail (# A32959, ThermoFisher Scientific)], mixed by vortexing, and incubated on ice for 20 min. The pellet suspensions were subjected to quick sonication (3 sec, 20% amplitude, FB505 Sonic Dismembrator, Fisher Scientific, Pittsburgh, PA), followed by incubation on ice for 10 min. The suspensions were centrifuged at 21,130 *x g* at 4°C for 10 min, and the supernatants were collected as nuclear lysates for the TF activation profiling array (#FA-1001, Signosis, Santa Clara, CA) according to the manufacturer’s instructions. Briefly, nuclear lysates (15 µg) were incubated with a TF probe mix containing known biotin-labeled probes/TF DNA-binding sequences. TF/DNA probe complexes were subsequently separated from free (unbound) probes using a spin column. The eluted TF/probe complexes were then denatured, followed by hybridization of detached eluted probes with a hybridization plate (a 96-well plate pre-coated with a specific complementary probe sequence corresponding to an individual TF in each well). The captured/bound DNA probes on the plate were washed, blocked, and incubated with a streptavidin-HRP conjugate and then with a substrate solution, followed by luminescence reading on a multimode plate reader (Bio-Tek Synergy HT, Winooski, VT). The values were normalized with corresponding Histone H3 expression levels for each group as detected by WB and quantitative analysis by an Image Lab software and expressed as relative light units (RLU). One well (TF: TR) for each group was used as background control, in which eluted probes were not added.

### 2.6 Immunofluorescence staining

293T cells were plated on a 24-well cell culture plate containing glass coverslips pre-coated with poly-L-lysine hydrobromide (#P6282, Sigma-Aldrich), transfected with pNL4-3 or pNL4-3 and pFlag-UbV15.1 (1:1 ratio), incubated for 16 hr, changed medium and continued to incubate for 48 hr. The cells were fixed in 4% paraformaldehyde for 15 min, permeabilized with 0.5% Triton-X100 for 10 min, blocked with PBS-BB buffer (1% BSA, 0.2% non-fat dry milk, and 0.3% Triton-X100) for 1 hr, incubated with mouse anti-p24 antibody 183-H12-5C (1:500, #ARP-1513, NIH HIV Reagent Program) at 4°C overnight, and then a goat anti-mouse IgG (H+L) secondary antibody, Alexa Fluor™ 555 (1:1000, #A-21424, Invitrogen) at RT in dark for 1 hr. The cells were stained with 1 µg/ml 4’,6-diamidino-2-phenylindole (DAPI) in the dark for 10 min. The cells were thoroughly washed with PBS between each step. The coverslips were mounted with Fluoromount-G^®^ (#0100-01, SouthernBiotech, Birmingham, AL) on the glass slides and sealed for imaging. The images were taken using a Nikon ECLIPSE 80*i* (BLAIS Microscope Company, Faribault, MN).

### 2.7 Data and statistical analysis

The GraphPad Prism 10 software was utilized for statistical analysis. The graphed data with only two sample groups were analyzed by unpaired *t-test*, and the data for multiple groups were analyzed by one-way *ANOVA* followed by either *Dunnett’s multiple comparisons test* (relevant to the reference group) or *Tukey’s multiple comparisons test (*for multiple group comparisons). The asterisk(s) for *p* values were: *, *p* < 0.05; **, *p* < 0.01; ***, *p* < 0.001; ****, *p* < 0.0001.

## 3 Results

### 3.1 Ectopic USP15 expression inhibited and UbV expression increased HIV-1 gene expression and virus production

**Figure 1:**
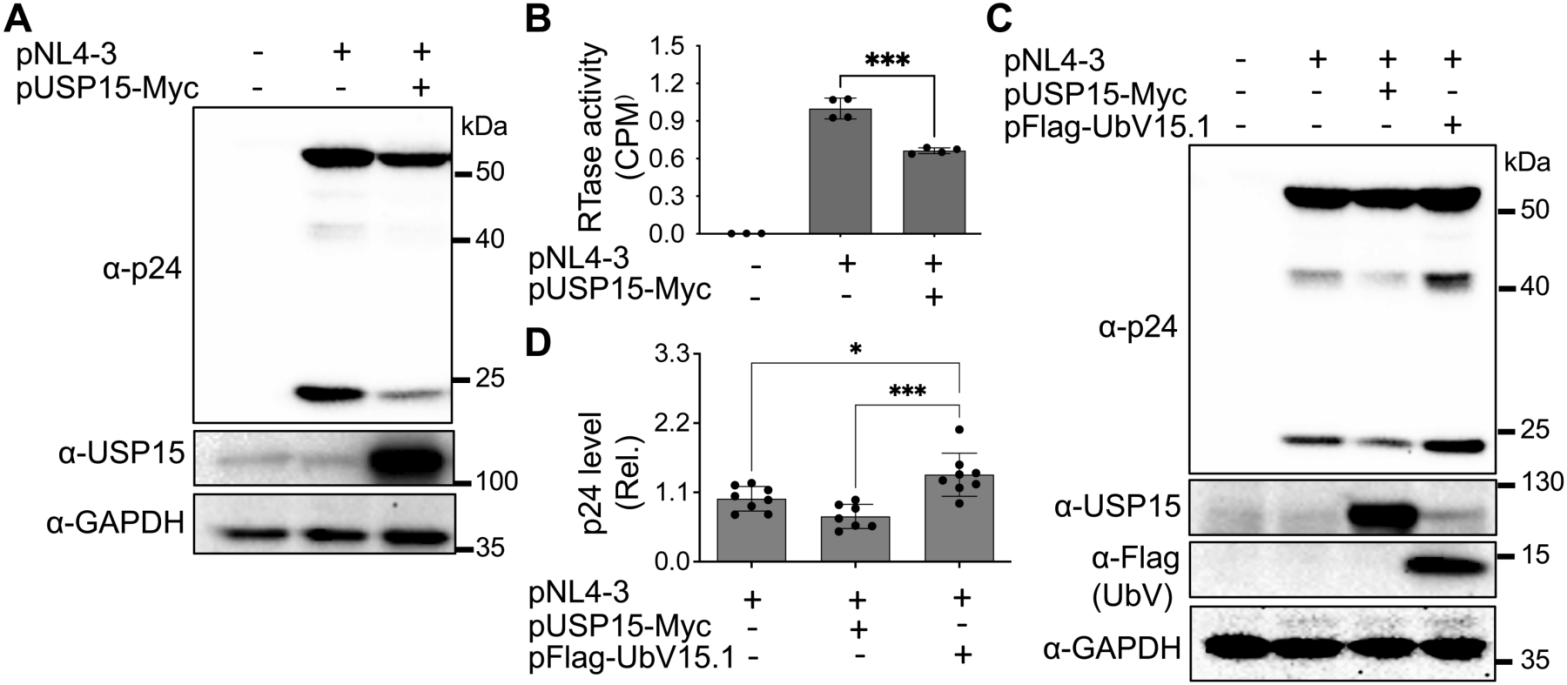
Effects of ectopic USP15 and UbV expression on gene expression and virus production of HIV-1 NL4-3. **(A, B)** 293T were transfected with pNL4-3, or pNL4-3 plus pUSP15-Myc (1:1 ratio). The cells were harvested for Western blotting against an anti-p24, anti-USP15, or anti-GAPDH antibody **(A)**, while the supernatants were collected for RTase activity assay **(B)**. The RTase activity was expressed as counts per minute (CPM), normalized with total cellular proteins, and compared to the pNL4-3 transfection control, which was set at 1.0. **(C, D)** 293T were transfected with pNL4-3, pNL4-3 plus pUSP15-Myc (1:1 ratio), or pNL4-3 plus pFlag-UbV15.1 (1:1 ratio). The cells were harvested for Western blotting against an anti-p24, anti-USP15, anti-Flag, or anti-GAPDH antibody **(C)**. p24 expression was quantitated by densitometry, normalized with GAPDH, and compared to the pNL4-3 transfection control, which was set at 1.0 **(D)**. pcDNA3 was included as a control and used to equalize the total DNA amounts among the transfections, and the cells only transfected with pcDNA3 were excluded from statistical analysis. The data were representative **(A, C)** and Mean ± SD of n ≥ 3 independent experiments **(B, D)**. *, p < 0.05; ***, p < 0.001.

**Figure 2:**
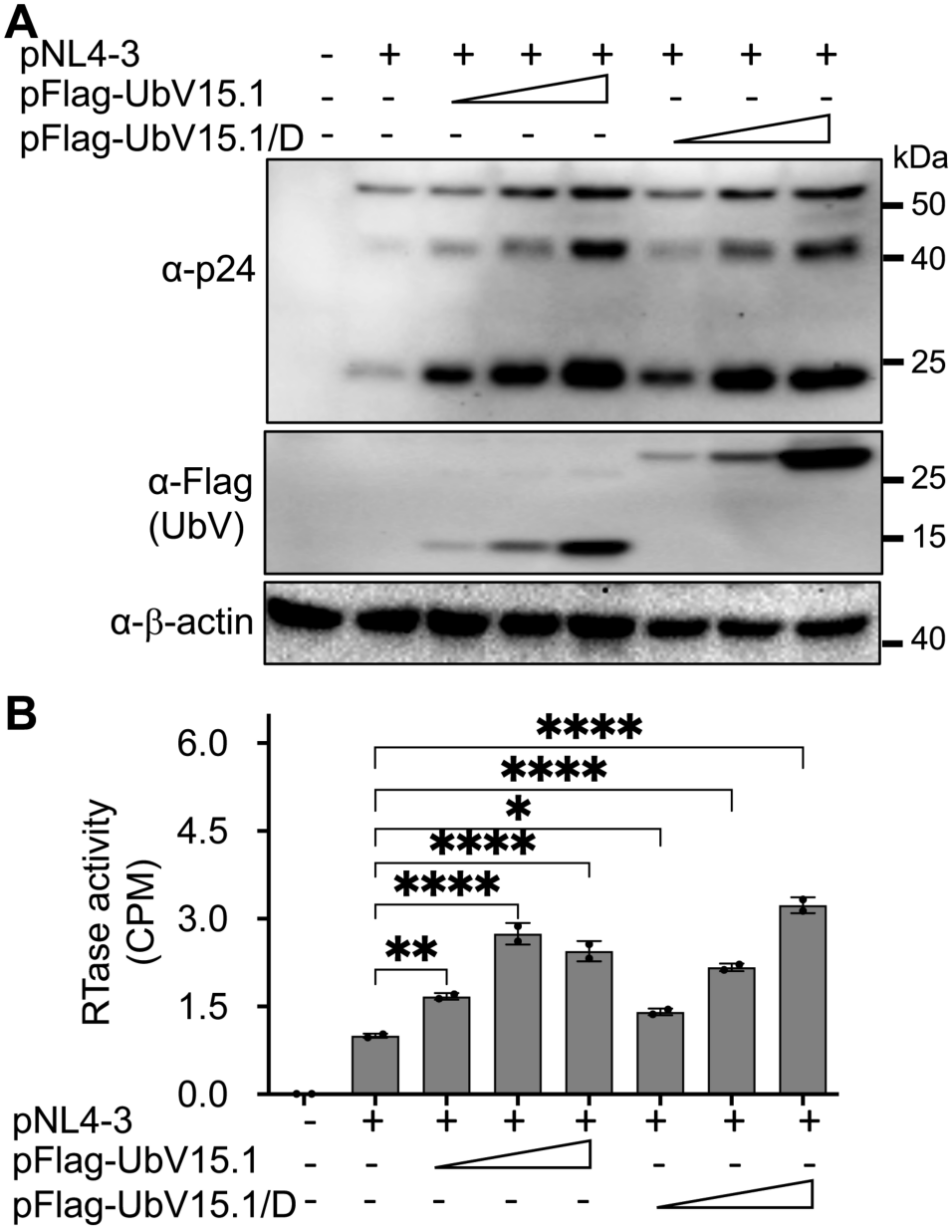
Relationship between ectopic UbV expression and HIV-1 gene expression and virus production of HIV-1 NL4-3. 293T were transfected with pNL4-3 (1 µg) or pNL4-3 plus increasing concentrations (0.5,1, and 2 µg) of pFlag-UbV15.1 or pFlag-UbV15.1/D. The cells were harvested for Western blotting against an anti-p24, anti-Flag, or anti-β-actin antibody **(A)**, while the supernatants were collected for RTase activity assay **(B)**. The RTase activity was expressed as counts per minute (CPM), normalized with total cellular proteins, and compared to the transfection with only pNL4-3. pcDNA3 was included as a control and used to equalize the total DNA amounts among the transfections, and the cells only transfected with pcDNA3 were excluded from statistical analysis. The data were Mean ± SD of duplicates (n = 2) and representative of three independent experiments. *, p < 0.05; **, p < 0.01; ****, p < 0.0001.

To gain a better understanding of the roles of USP15 in HIV infection and pathogenesis, we first wanted to ascertain the inhibitory effect of USP15 on HIV-1 gene expression and viral production. Thus, we overexpressed USP15 in the presence of HIV and determined viral gene expression and virus production. HIV-1 gene expression, measured by Western blotting (WB) against HIV-1 p24 showed a remarkable decrease in cells expressing exogenous USP15 when compared to the control without exogenous USP15 (**Figure 1A**). As expected, there was a significant decrease in HIV-1 production, measured by the HIV RTase activity of the culture medium, in the cells expressing exogenous USP15 when compared to the control without exogenous USP15 (**Figure 1B**). To rule out the possibility that USP15-induced inhibition of HIV-1 gene expression and virus production was not due to any artifacts often associated with gene overexpression, we took advantage of USP15 UbV, which were recently developed to be USP15 inhibitors using the phage display combination library screening (Teyra et al., 2019). We first used UbV15.1, a USP15-selective UbV targeting the catalytic and adaptor domain of USP15 and determined if its overexpression would have the opposite effects. In contrast to USP15 overexpression, UbV15.1 overexpression showed significantly more p24 expression compared to the samples with or without ectopic USP15 expression (**Figures 1C, D**). Immunofluorescence showed increased p24 staining and intensity in cells expressing UbV15.1 (**Supplementary Figure S1**). The enhancement effects of the USP15 UbV on HIV-1 gene expression and virus production were further demonstrated in a dose-dependent manner and using UbV15.1/D, a USP15-specific UbV targeting both the domain-specific USP (DUSP) and catalytic domains of USP15 (**Figure 2**).

**Figure 3:**
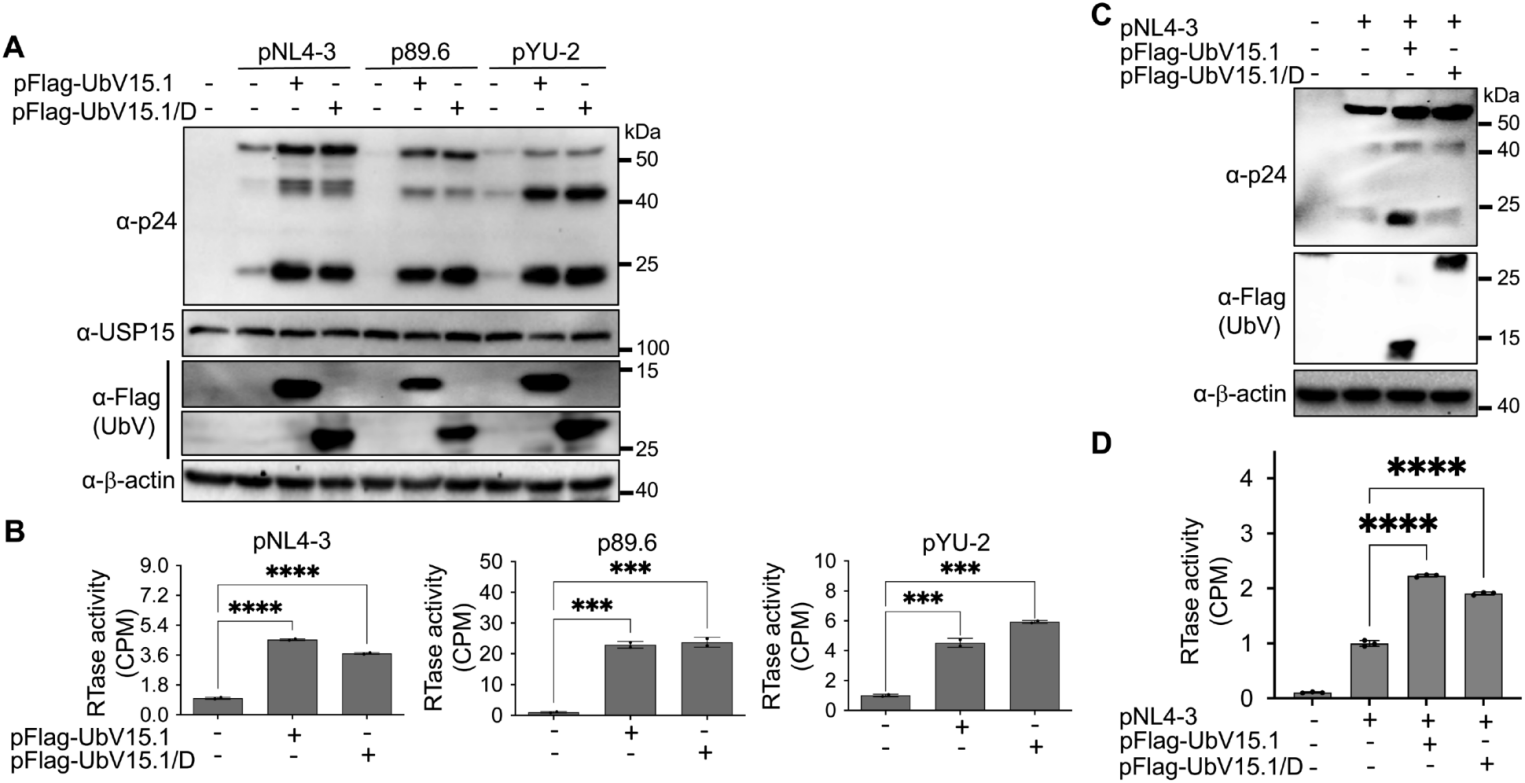
Effects of ectopic UbV expression on gene expression and virus production of HIV-1 NL4-3, 89.6, and YU-2. **(A, B)** 293T were transfected with one of the HIV plasmids (1 µg) pNL4-3, p89.6, and pYU-2, or one of the HIV plasmids pNL4-3, p89.6, and pYU-2 plus one of the UbV plasmids pFlag-UbV15.1 and pFlag-UbV15.1/D (2 µg). The cells were harvested for Western blotting against an anti-p24, anti-USP15, anti-Flag, or anti-β-actin antibody **(A)**, while the supernatants were collected for RTase activity assay **(B)**. The RTase activity was expressed as counts per minute (CPM), normalized with total cellular proteins, and compared to the transfection with indicated HIV plasmids only. pcDNA3 was included as a control and used to equalize the total DNA amounts among all transfections, and the cells only transfected with pcDNA3 were excluded from statistical analysis. **(C, D)** Jurkat were transfected with pNL4-3 or pNL4-3 plus one of the UbV plasmids pFlag-UbV15.1 and pFlag-UbV15.1/D (1:1 ratio). The cells were harvested for Western blotting against an anti-p24, anti-Flag, or anti-β-actin antibody **(C)**, while the supernatants were collected for RTase activity assay **(D)**. The RTase activity was expressed as counts per minute (CPM), per 1 µg cellular proteins, and compared to the transfection with pNL4-3 only. pcDNA3-GFP was included as a control and used to equalize the total DNA amounts among the transfections, and the cells only transfected with pcDNA3-GFP were excluded from statistical analysis. The data were representative and Mean ± SD of duplicates (n = 2, **B**) or triplicates (n = 3, **D**). ***, p < 0.001; ****, p < 0.0001.

To determine if these two USP15 UbV would have similar effects on gene expression and virus production of other HIV-1 isolates, we performed similar experiments with HIV-1 89.6 (Smyth et al., 1998) and HIV-1 YU-2 (Rana et al., 1997). Similar to pNL4-3, both 89.6 and YU-2 showed increased p24 expression (**Figure 3A**) and significant virus production (**Figure 3B**) in cells expressing either UbV inhibitor. In addition, a comparable dose-dependent enhancement kinetics of HIV-1 gene expression was noted among the HIV-1 isolates (**Supplementary Figure S2**). Furthermore, we also performed similar experiments in CD4+ T lymphocytes Jurkat. Similar to 293T, Jurkat expressing either USP15 UbV had significantly higher p55 and p24 expression and virus production compared to the control (**Figures 3C, D**). These results collectively demonstrated the inhibitory effects of USP15 on HIV-1 gene expression and virus production and suggest the important roles of USP15 in HIV infection and pathogenesis.

### 3.2 Ectopic USP15 expression repressed HIV-1 transcription, whereas UbV expression increased HIV-1 transcription

**Figure 4:**
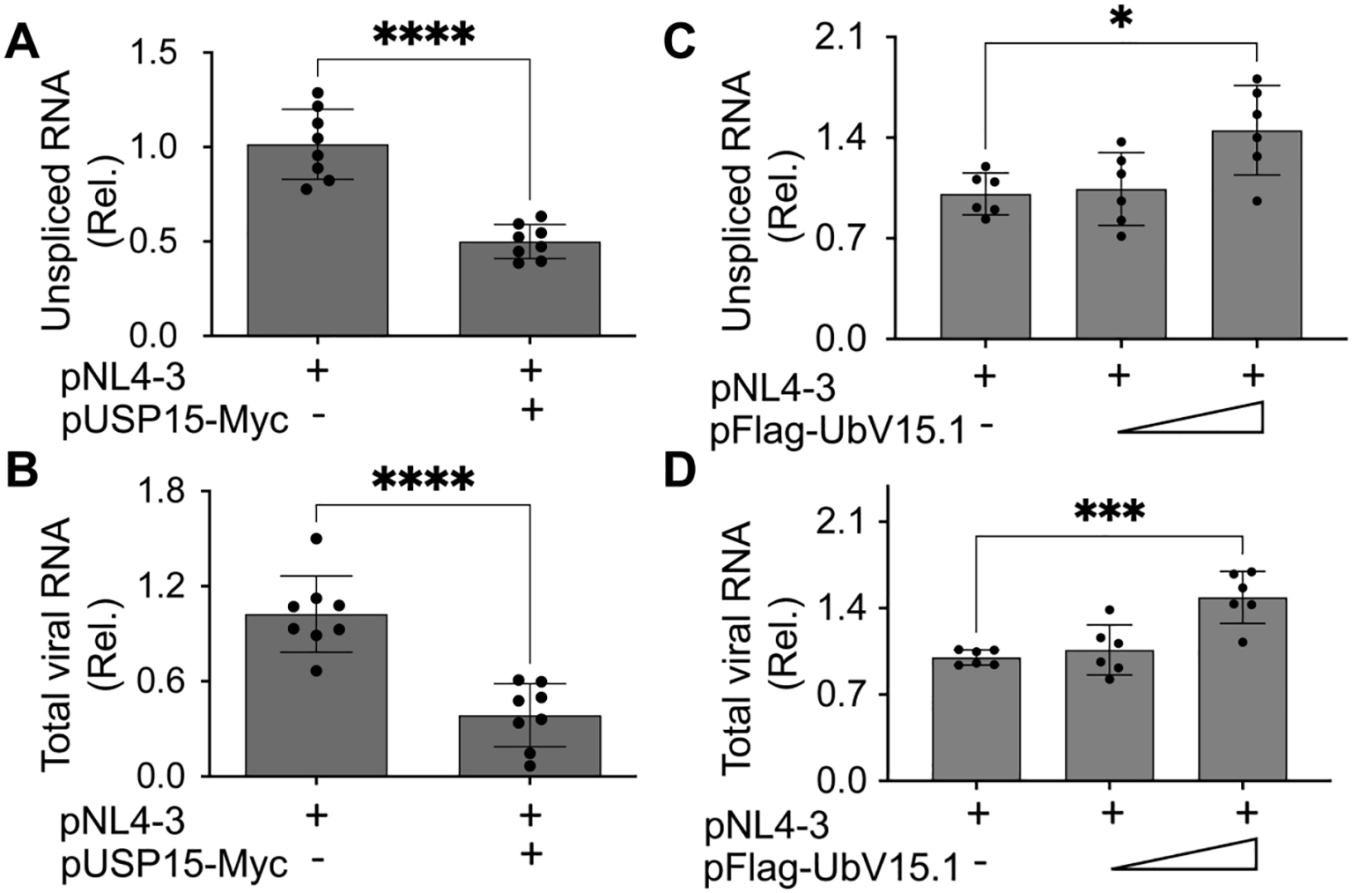
Effects of ectopic USP15 and UbV expression on HIV-1 gene transcription. 293T were transfected with pNL4-3, pNL4-3, and pUSP15-Myc (1:1 ratio, **A, B**), or pNL4-3 plus pFlag-UbV15.1 (1:0.5 and 1:1 ratio, **C, D**). The cells were collected for total RNA isolation and qRT-PCR using primers specific for unspliced HIV RNA (gag-pol) **(A, C)** or total HIV RNA **(B, D)**. Unspliced and total HIV RNA levels were calculated, normalized to the internal control GAPDH, and expressed as relative target quantity minimum (RQ Min) and maximum (RQ Max) of the replicates in each group and compared to the transfection with pNL4-3 only, which was set at 1.0. The data were Mean ± SD of RQ Min and Max of multiple replicates of four independent experiments **(A, B)** or three independent experiments **(C, D)**. *, p < 0.05; ***, p < 0.001; ****, p < 0.0001.

Next, we investigated if HIV-1 transcription would contribute to the changes in HIV-1 gene expression and virus production by USP15 or its UbV inhibitors. To this end, we first transfected 293T with pNL4-3 and with or without pUSP15-Myc, isolated total RNA, and performed qRT-PCR for both unspliced HIV RNA and total HIV RNA. There was a significant decrease in both unspliced HIV RNA (**Figure 4A**) and total HIV RNA (unspliced plus spliced RNA) (**Figure 4B**) in cells expressing exogenous USP15 when compared to the control without exogenous USP15. We then performed similar experiments with pFlag-UbV15.1 and found a trend of increase in unspliced (**Figure 4C**) and total HIV RNA (**Figure 4D**), which only achieved a significant difference at the higher concentration of UbV15.1. These results provided the first evidence to support the regulatory roles of USP15 in HIV-1 transcription.

### 3.3 Changes in expression and activities of transcription factors resulting from ectopic USP15 expression

**Figure 5:**
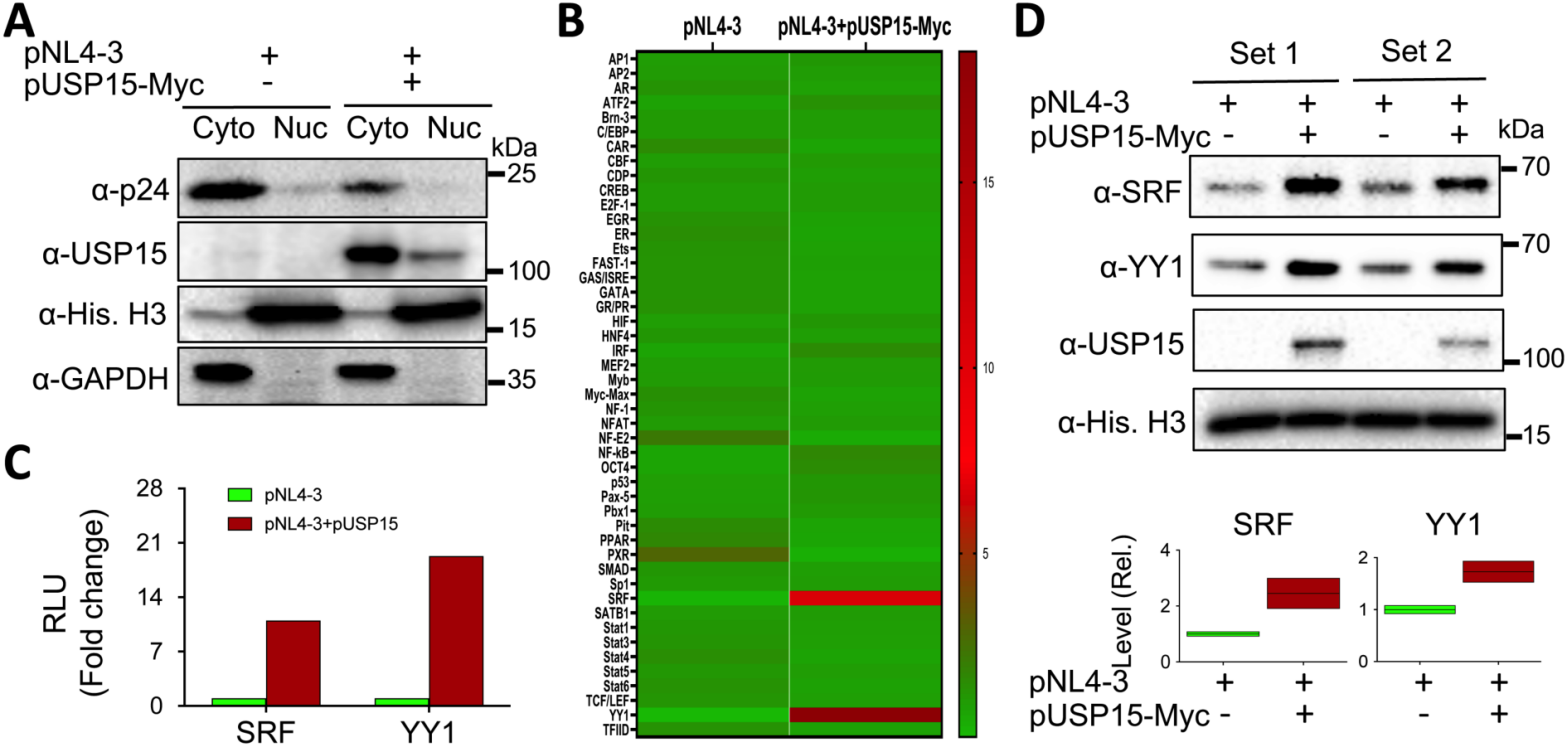
Effects of ectopic USP15 expression on expression and activities of transcription factors. 293T were transfected with pNL4-3 or pNL4-3 plus pUSP15-Myc (1:1 ratio). pcDNA3 was included to equalize the total DNA amounts among the transfections. The cells were harvested for nuclear and cytoplasmic lysates, followed by Western blotting using an anti-p24, anti-USP15, anti-Histone H3, or anti-GAPDH **(A)**, or the TF activation profiling array **(B)**. Luminescence readings from the array were quantitated, normalized to the internal controls, and expressed in relative light units (RLU). A heatmap was constructed based on the differences in the RLU between the two groups, in which the Chartreuse green color at the bottom represents the lowest activity level, and the crimson red color at the top represents the highest activity level. The RLU differences of SRF and YY1 activities from the array were presented **(C)**, while the nuclear lysates from two independent experiments (Sets 1 & 2) were analyzed for SRF and YY1 protein levels by Western blotting using an anti-SRF, anti-YY1, anti-USP15, or anti-Histone H3 antibody, followed by densitometry quantitation **(D)**.

A number of cellular transcription factors have been identified to regulate, positively or negatively, HIV-1 transcription and latency through direct binding and recruitment to DNA binding sites within the HIV-1 LTR promoter and/or indirectly through interaction with other cellular/viral factors (Pereira et al., 2000; Ruelas and Greene, 2013; Ne et al., 2018; Nchioua et al., 2020). Thus, we then determined the changes in both the expression and activity of cellular transcription factors in the presence of exogenous USP15 expression using a transcription factor activation profiling array, which is a DNA/transcription factor-binding-based luminescence assay and allows simultaneous detection of 48 cellular transcription factors. We transfected 293T with pNL4-3 and with or without pUSP15-Myc, prepared nuclear/cytoplasmic lysates, and performed WB. The use of nuclear marker Histone H3 and cytoplasmic marker GAPDH for Western blotting confirmed minimal cross-contamination between the nuclear/cytoplasmic lysates while a remarkable decrease of p24 in the cytoplasmic lysates of cells expressing exogenous USP15 was also verified (**Figure 5A**). Then, we performed the array using the nuclear lysates. Comparisons of transcription factors between these two groups, shown in the heatmap, revealed four transcription factors with the noticeable changes, Yin-Yang 1 (YY1) and serum response factor (SRF) up-regulated and pregnane X receptor (PXR) and nuclear factor erythroid-derived 2 (NF-E2) down-regulated in cells expressing exogenous USP15 compared to the control without exogenous USP15 (**Figure 5B**). The quantitative analysis of the luminescence reading values showed that YY1 and SRF had the most changes, up-regulation by approximately 19 folds and 11 folds, respectively (**Figure 5C**). To validate these findings further, we performed WB for YY1 and SRF expression in the nuclear lysates that were used for the array (set 1) and the nuclear lysates prepared from other independent experiments (set 2). We confirmed noticeable up-regulation of YY1 and SRF in the nucleus of cells expressing exogenous USP15 compared to the control without exogenous USP15 (**Figure 5D**). The differences in fold changes between the array and the Western blotting are likely due to the fact that the former is to measure both the expression and DNA-binding activity while the latter is to only measure the expression.

### 3.4 UbV expression decreased the nuclear YY1 protein level

**Figure 6:**
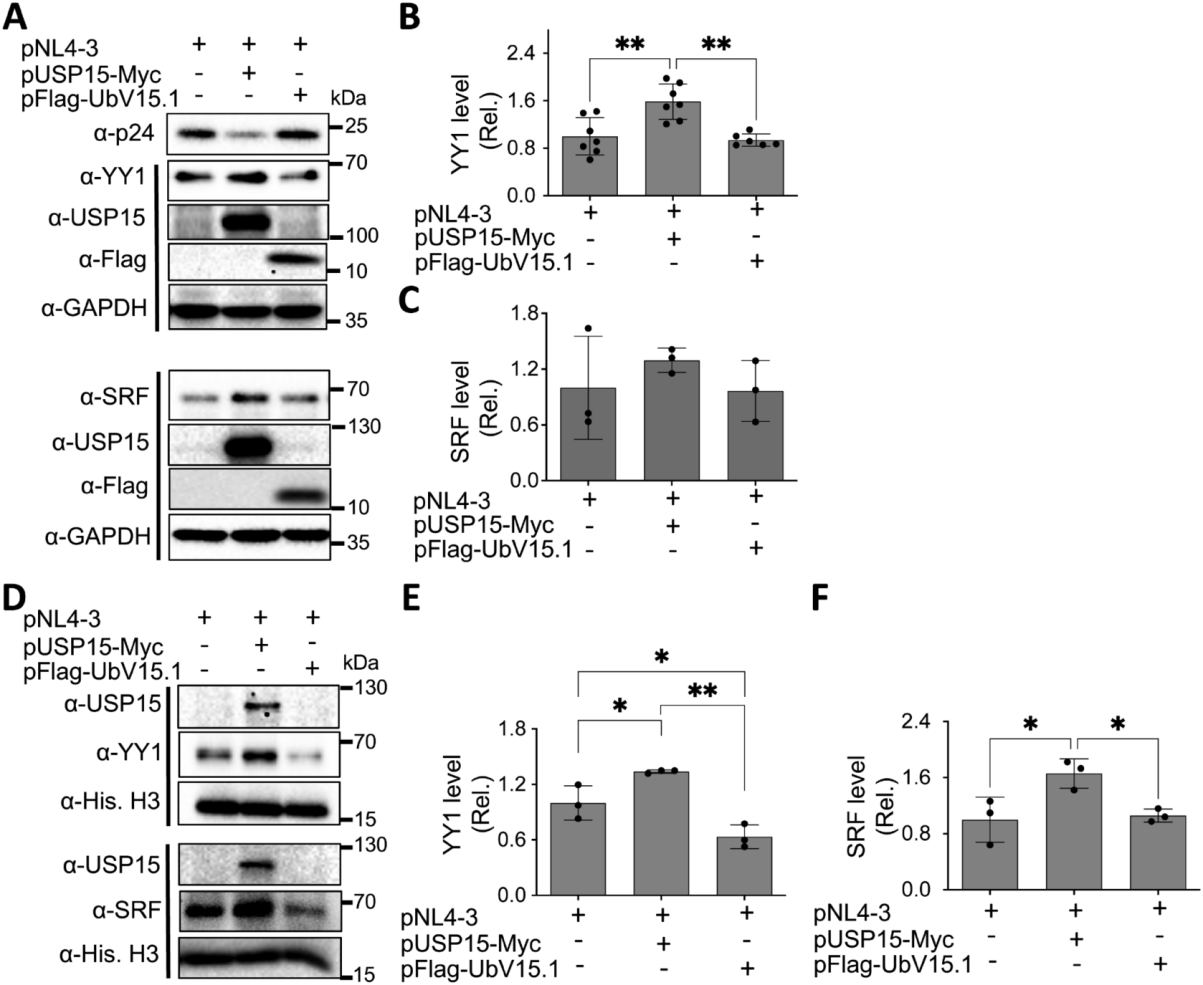
Effects of ectopic UbV expression on YY1 and SRF protein levels. 293T were transfected with pNL4-3, pNL4-3 plus pUSP15-Myc (1:1 ratio) or pNL4-3 plus pFlag-UbV15.1 (1:1 ratio). pcDNA3 was included to equalize the total DNA amounts among the transfections. The cells were harvested for whole cell lysates **(A–C)** or nuclear lysates **(D–F)**, followed by Western blotting **(A, D)** against an anti-p24, anti-YY1, anti-SRF, anti-USP15, anti-Flag, anti-GAPDH, or anti-Histone H3. The relative protein levels (Rel.) of YY1 **(B, E)** and SRF **(C, F)** were quantified by densitometry and normalized with GAPDH **(B, C)** or Histone H3 **(E, F)**. The data were representative of three independent experiments **(A, D)** and Mean ± SD of multiple replicates (n ≥ 6, **B**; n = 3, **C, E, F**) *, p < 0.05; **, p < 0.01.

Many transcription factors reside in the cytoplasm, become translocated to the nucleus in response to appropriate signals or stimuli, and regulate gene expression (Liu et al., 2018). Thus, we then determined if USP15 UbV inhibitors would alter subcellular distribution of nuclear YY1 and SRF proteins. We transfected 293T with pNL4-3 and with or without pUSP15-Myc or pFlag-UbV15.1, prepared whole cell lysates and nuclear lysates, and performed WB. The whole cell lysates of cells expressing exogenous USP15 displayed significantly more YY1 and a trend of increase in SRF compared to the cells without exogenous USP15 (**Figures 6A–C**). In comparison, UbV15.1 expression resulted in a noticeable decrease in YY1 and a trend of decrease in SRF compared to USP15 expression, but there were no differences in relative levels of both YY1 and SRF between cells expressing UbV15.1 and the cells transfected with pNL4-3 only. In nuclear lysates, ectopic USP15 expression showed significant increases in both YY1 and SRF when compared to the control transfection with pNL4-3 only, while UbV15.1 expression had significant decreases in both YY1 and SRF when compared to the cells expressing exogenous USP15 (**Figures 6D–F**). In addition, there were significant differences in YY1 but no differences in SRF between UbV15.1 expression and the control with pNL4-3 only. Consistent with the notion that YY1 is a negative regulator of HIV-1 transcription (Margolis et al., 1994; Romerio et al., 1997; Coull et al., 2000; He and Margolis, 2002; Bernhard et al., 2013), our findings that ectopic USP15 expression up-regulated YY1 while USP15 UbV expression decreased nuclear YY1 protein level raised the possibility that YY1 might play a crucial role in the inhibitory function of USP15 in HIV transcription.

### 3.5 No apparent changes of YY1 mRNA expression by ectopic USP15 and UbV expression

**Figure 7:**
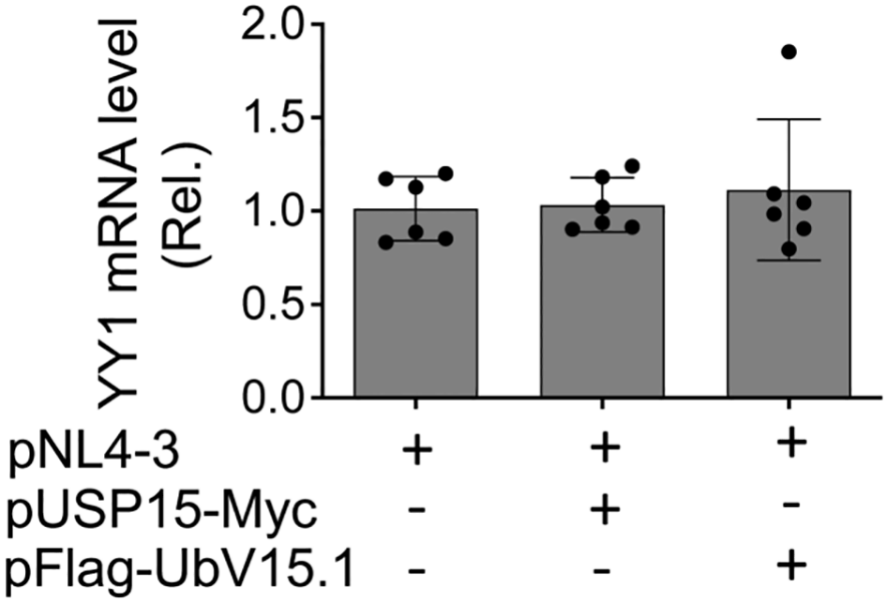
Effects of ectopic USP15 and UbV expression on YY1 mRNA level. 293T were transfected with pNL4-3, pNL4-3 plus pUSP15-Myc (1:1 ratio) or pNL4-3 plus pFlag-UbV15.1 (1:1 ratio). pcDNA3 was included to equalize the total DNA amounts among the transfections. The cells were harvested for total RNA isolation and qRT-PCR. YY1 mRNA levels were calculated based on the RQ Min and Max of the replicates, normalized to the internal control GAPDH, and compared to the transfection with pNL4-3 only, which was set at 1.0. The data were Mean ± SD of RQ Min and Max of multiple replicates of three independent experiments.

To determine if ectopic USP15 and UbV expression could also alter YY1 transcription and subsequently contribute to changes in YY1 protein expression, we transfected 293T with pNL4-3 and with or without pUSP15-Myc or pFlag-UbV15.1, isolated total RNA, and performed qRT-PCR for the YY1 mRNA level. The results showed that neither ectopic USP15 expression nor UbV expression resulted in any apparent changes in YY1 mRNA expression when compared to the control or to each other (**Figure 7**), indicating that changes of YY1 protein expression by ectopic USP15 and UbV expression are unlikely to occur at the transcription level, and rather at the post-translational level.

### 3.6 Ectopic USP15 and UbV expression altered YY1 ubiquitination

**Figure 8:**
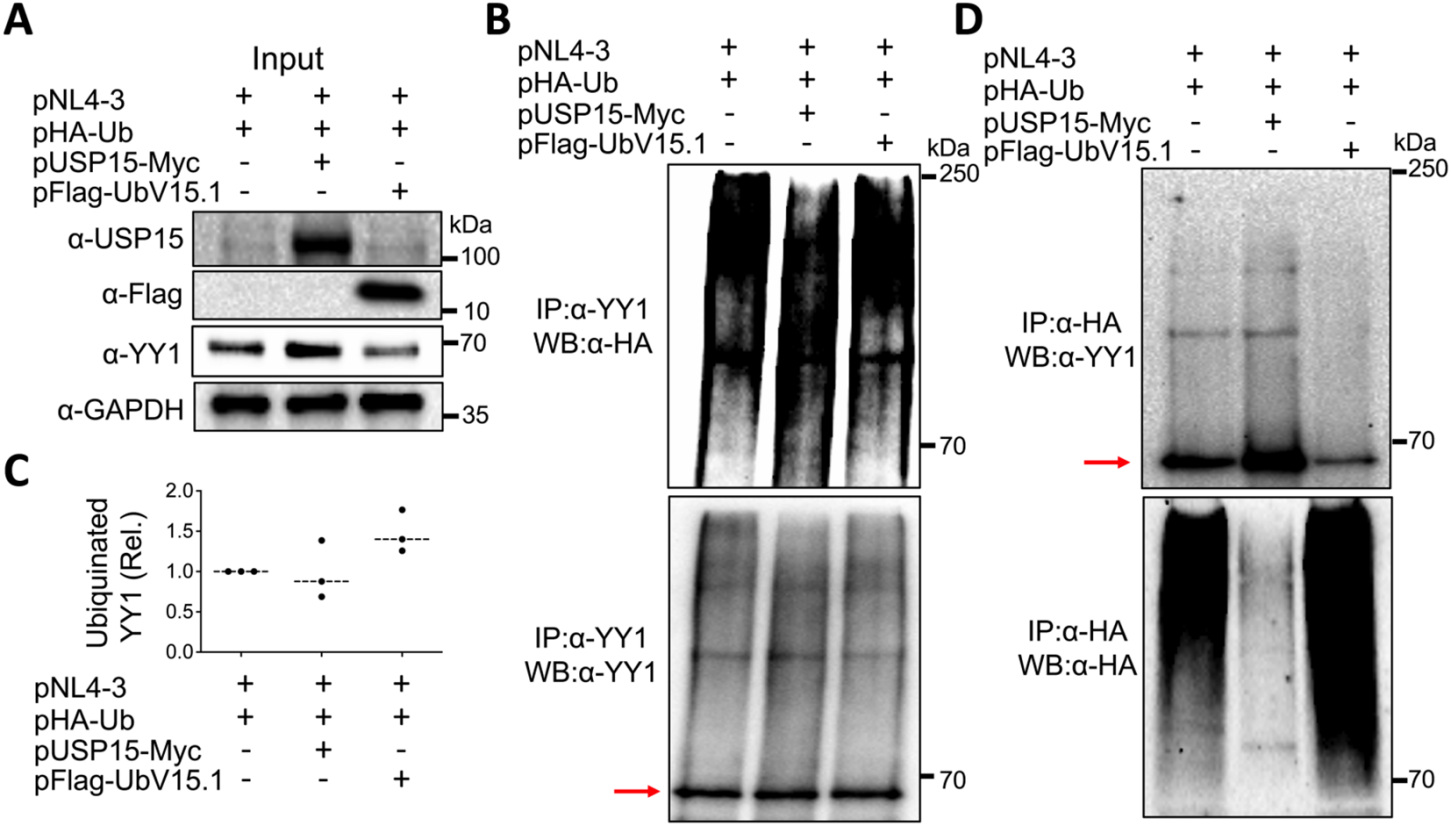
Effects of ectopic USP15 and UbV expression on YY1 ubiquitination. 293T were transfected with pNL4-3, pHA-Ub, and either pUSP15-Myc or pFlag-UbV15.1 (1:1:1 ratio). pcDNA3 was included to equalize the total DNA amounts among the transfections. The cells were treated with carfilzomib (20 nM) for 24 hr and harvested for whole cell lysates, followed by Western blotting (WB) against an anti-USP15, anti-Flag, anti-YY1, or anti-GAPDH antibody **(A)**, or immunoprecipitation (IP) against an anti-YY1 antibody and WB against an anti-HA or anti-YY1 antibody **(B)**. Relative YY1 ubiquitination **(C)** was calculated by dividing the total ubiquitinated YY1 protein (upper panel, **B**) by the total input YY1 protein **(A)** and normalized to the input GAPDH **(A)**. IP against an anti-HA antibody and WB against an anti-YY1 or anti-HA antibody were also performed **(D)**. The data were representative of three independent experiments **(A, B, D)** and Mean ± SD of triplicates (n = 3, **C**). Red arrow: YY1.

Post-translational modifications are crucial in altering and regulating the function of the proteins. Among them are ubiquitination and deubiquitination, which affect the stability of proteins through the ubiquitin-proteasome pathway and as a result, the protein homeostasis and biological functions (Wang and Wang, 2021; Lee et al., 2023). Thus, we then investigated the possibility that the deubiquitinase function of USP15 or the inhibition of USP15 deubiquitinating activity by its UbV inhibitors might target YY1 for deubiquitination/ubiquitination and, therefore, YY1 protein stabilization/degradation and impact HIV-1 transcription. To address this possibility, we transfected 293T with pNL4-3, HA-tagged ubiquitin plasmid pHA-Ub, and with or without pUSP15-Myc or pFlag-UbV15.1, treated with a proteasome inhibitor carfilzomib 24 hr, prepared whole cell lysates, and performed WB, or IP followed by WB. Direct WB of whole cell lysates confirmed USP15, UbV expression, and increased YY1 protein expression by exogenous USP15 and decreased YY1 protein expression by UbV15.1 even in the presence of Carfilzomib treatment (**Figure 8A**). IP against an anti-YY1 antibody followed by WB against an anti-HA antibody showed less poly-ubiquitinated YY1 protein/smear by USP15 and more poly-ubiquitinated YY1 protein/smear by UbV15.1 compared to the control without USP15 or UbV15.1 (upper panel, **Figures 8B, C**), while IP against an anti-YY1 antibody followed by WB against the same anti-YY1 antibody detected a similar level of YY1 protein (lower panel, **Figure 8B**). Interestingly, IP against an anti-HA antibody followed by WB against an anti-YY1 antibody showed one dominant and increased mono-ubiquitinated YY1 protein by USP15 and a decreased mono-ubiquitinated YY1 protein by UbV15.1 compared to the control without USP15 or UbV15.1 (upper panel, **Figure 8D**), whereas IP against an anti-HA antibody followed by WB against the same anti-HA antibody showed noticeably less ubiquitinated cellular proteins/smear by USP15 and more ubiquitinated cellular proteins/smear by UbV15.1 compared to the control without USP15 or UbV15.1 (lower panel, **Figure 8D**). The apparent counterintuitive findings were likely due to stabilization and accumulation of YY1 protein by ectopic USP15 expression giving rise to seemingly more ubiquitinated YY1 protein in cell expressing exogenous USP15. Nevertheless, these results demonstrated that ectopic USP15 and UbV expression led to changes of YY1 ubiquitination/deubiquitination and stability/degradation. These results also suggest that USP15 may target other cellular proteins in addition to YY1.

### 3.7 USP15 interaction with YY1

**Figure 9:**
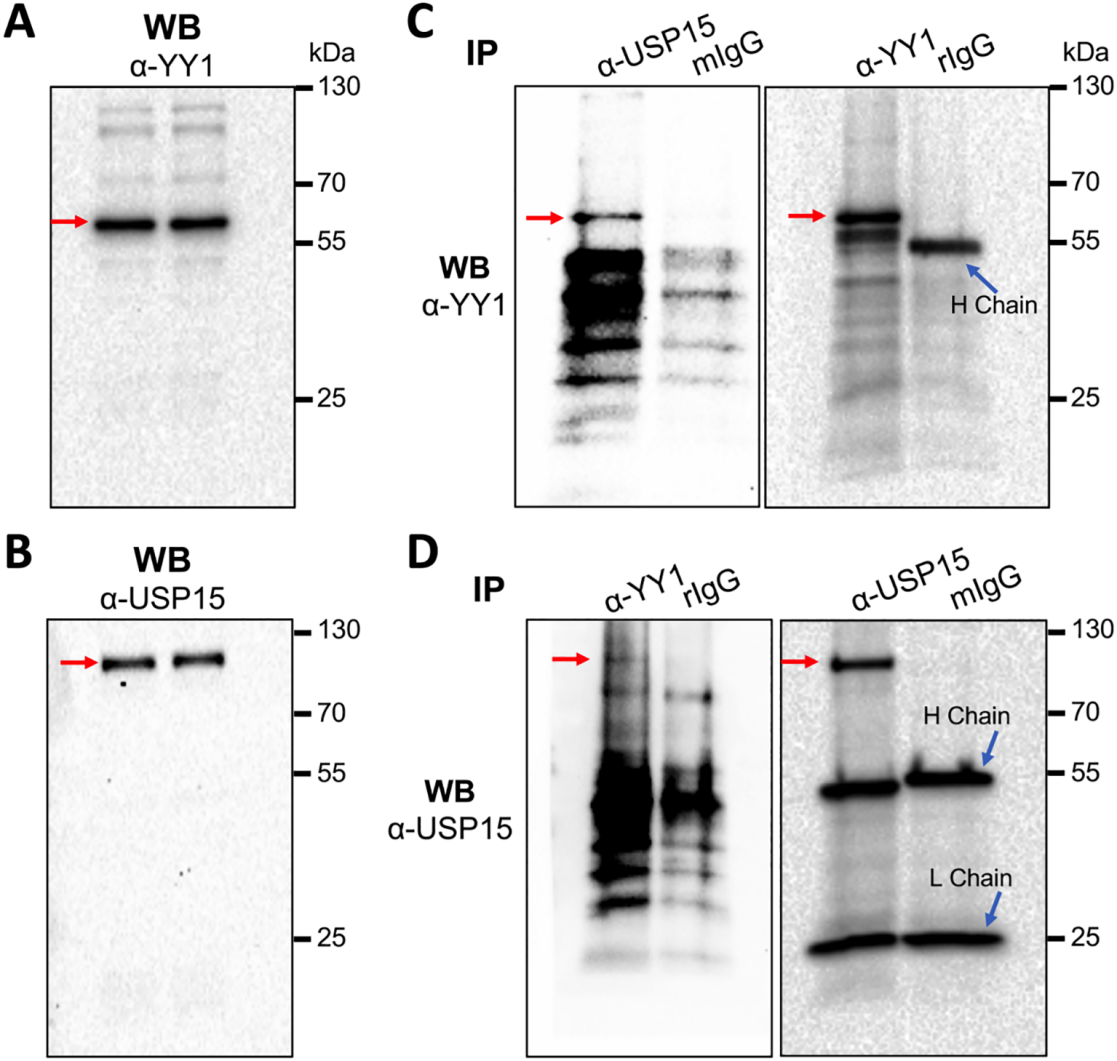
Complex formation between endogenous USP15 and YY1. 293T were harvested for whole cell lysates, followed by WB against an anti-YY1 antibody **(A)**, or anti-USP15 antibody **(B)**, or IP against an anti-USP15 or anti-YY1 antibody and WB against an anti-YY1 antibody **(C)**, or IP against an anti-YY1 or anti-USP15 antibody and WB against an anti-USP15 antibody **(D)**. A mouse isotype-matched IgG (mIgG) and a rabbit IgG (rIgG) were included as the controls for mouse anti-USP15 antibody and rabbit anti-YY1 antibody, respectively. YY1 and USP15 proteins were marked by red arrows.

USP15 interaction with YY1 would be imperative for YY1 to serve as a substrate of USP15 to be deubiquitinated. Thus, we next determined if there would be any interaction between endogenous USP15 and YY1. Direct WB of whole cell lysates confirmed expression of endogenous YY1 (**Figure 9A**) and USP15 (**Figure 9B**). We then performed IP against an anti-USP15 or anti-YY1 antibody, followed by WB against the same YY1 antibody and detected YY1 in both USP15- and YY-1 immunoprecipitates, not in the IgG controls (**Figure 9C**). Similarly, we performed IP against an anti-YY1 or anti-USP15 antibody, followed by WB against the same USP15 antibody and detected USP15 in both YY1- and USP15 immunoprecipitates, not in the IgG controls (**Figure 9D**). These results support the possibility that USP15 forms a complex with YY1.

### 3.8 YY1 knockdown rescued the inhibitory effects of USP15 on HIV-1 gene expression and virus production, while USP15 knockdown led to decreased YY1 and increased HIV-1 gene expression and virus production

**Figure 10:**
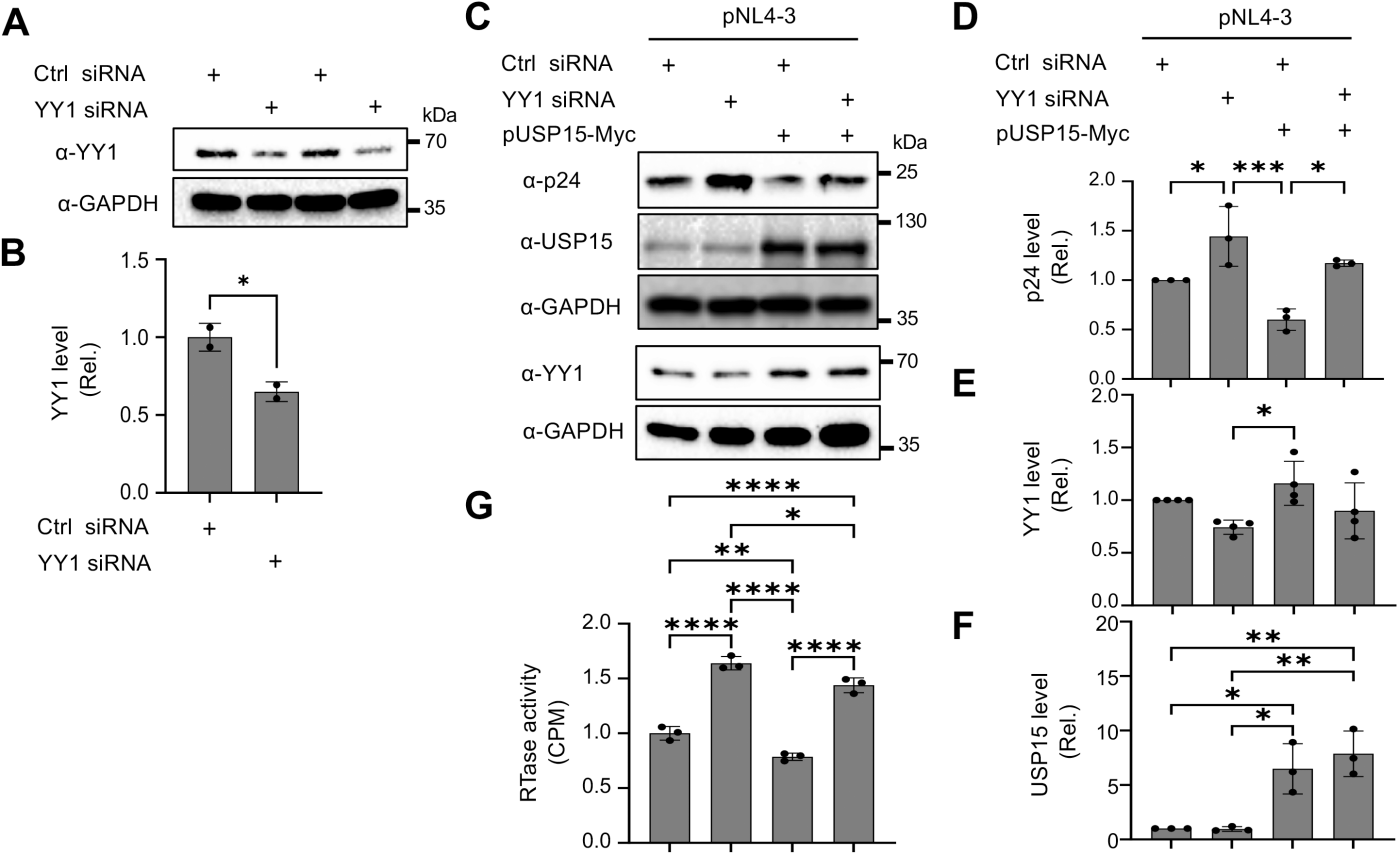
Effects of YY1 knockdown on HIV-1 gene expression and viral production in presence of ectopic expression of USP15. 293T were plated in a 6-well plate, cultured for 24 hr, transfected with control (Ctrl) siRNA (100 nM) or YY1 siRNA (100 nM) for 48 hr **(A, B)**. The cells were then replated, cultured for 24 hr, transfected with 1 µg pUSP15 and 1 µg pNL4-3, and cultured for 48 hr **(C–G)**. pcDNA3 was included to equalize transfected DNA among the transfections. **(A, B)** YY1 knockdown by YY1 siRNA by Western blotting prior to pUSP15 and pNL4-3 transfection **(A)**. The relative level was determined by densitometry, normalized to GAPDH, and expressed in fold changes over the control siRNA **(B)**. (**C–F**). p24, USP15, and YY1 expression following YY1 siRNA, pUSP15, and pNL4-3 transfection by Western blotting **(C)**. The replated YY1 knockdown cells were transfected with pNL4-3, with or without pUSP15-Myc (1:1 ratio) and incubated for 48 hours. The cells were collected for Western blotting and probed with antibodies against p24, USP15, YY1, and GAPDH **(C)**. The relative level was determined by densitometry, normalized to GAPDH, and expressed in fold changes over the first control siRNA **(D–F)**. The culture supernatants were collected for RTase activity assay, which was expressed in counts per min (CPM) per ml supernatant, and presented in fold changes over the first control siRNA **(G)**. The statistical analysis was performed using *unpaired t-test* **(B)** or one-way *ANOVA*, followed by *Tukey’s multiple comparisons test* for comparisons between groups **(D–G)**. The data were representative of four independent experiments **(C–G)** and Mean ± SD of multiple replicates (n = 2, **B**; n = 3, **D, F, G**; n = 4, **E**). *, p < 0.05; **, p < 0.01; ***, p < 0.001; ****, p < 0.0001.

**Figure 11:**
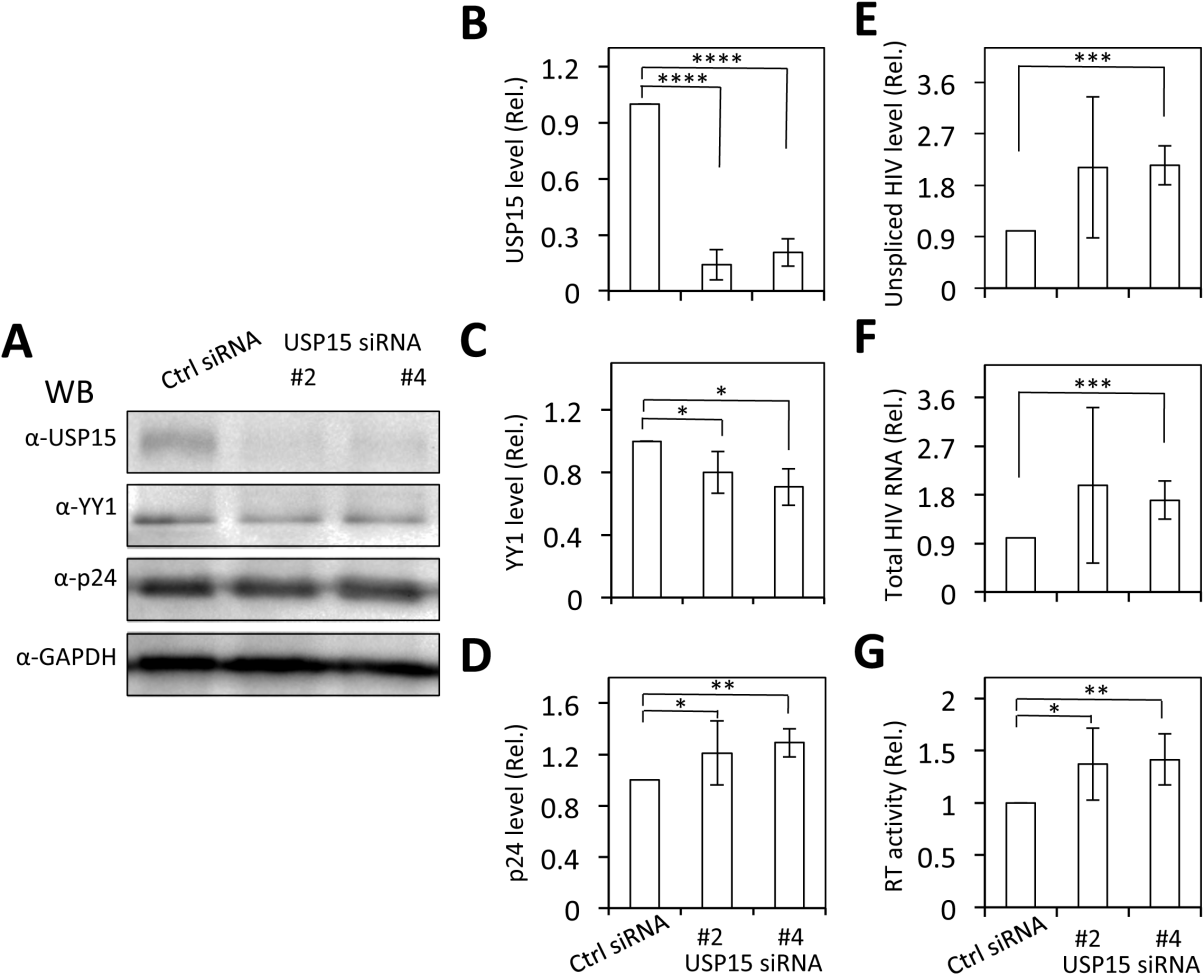
Effects of USP15 knockdown on YY1 expression and HIV-1 gene expression and viral production. 293T were plated in a 6-well plate, cultured for 24 hr, transfected with On-Targetplus non-targeting control (Ctrl) siRNA (100 nM) or On-Targetplus USP15 siRNA #2 and #4 (100 nM) for 48 hr. The cells were then replated, cultured for 24 hr, transfected with 2 µg pNL4-3, and cultured for 48 hr. The cells were harvested. Two third of the cells were used for cell lysates and Western blotting against USP15, YY1, p24, and GAPDH **(A)**. The relative level was determined by densitometry, normalized to GAPDH, and expressed in fold changes over the siRNA control **(B–D)**. One third of the cells were used for total RNA isolation and qRT-PCR. The relative level was normalized to the internal control GAPDH and expressed in fold changes over the siRNA control **(E, F)**. The culture supernatants were collected for RTase activity assay, which was expressed in counts per min (CPM) per ml supernatant and presented in fold changes over the siRNA control **(G)**. The statistical analysis was performed using one-way *ANOVA*, followed by *Tukey’s* *multiple comparisons test* for comparisons between groups **(B–G)**. The data were representative of three independent experiments and Mean ± SD of three replicates (n = 3). *, p < 0.05; **, p < 0.01; ***, p < 0.001; ****, p < 0.0001.

To ascertain the involvement of YY1 in USP15-mediated HIV-1 inhibition, we used the siRNA knockdown strategy and determined if the reduction in YY1 expression would rescue the inhibitory effect of USP15 on HIV-1 gene expression and replication. First, we transfected 293T with YY1 siRNA and confirmed a significant reduction in YY1 expression in YY1 siRNA transfected samples compared to the siRNA control (**Figures 10A, B**). Then, we replated these siRNA transfected cells, transfected with pNL4-3 and pUSP15-Myc, and determined effects of YY1 knockdown on HIV-1 gene expression and production. We showed that YY1 siRNA knockdown led to a remarkable increase in HIV-1 p24 gene expression compared to the control siRNA (**Figures 10C, D**). USP15 inhibited p24 expression, but the inhibition was significantly diminished by the YY1 siRNA knockdown. In the meantime, we confirmed YY1 and USP15 expression and the changes in YY1 induced by USP15 expression (**Figures 10C–F**). In parallel, YY1 siRNA knockdown led to a significant increase in HIV-1 production, while USP15 expression inhibited HIV-1 production (**Figure 10G**). In agreement with the p24 expression, USP15 inhibited HIV-1 production, but the inhibition was significantly diminished by the YY1 siRNA knockdown. To further ascertain the relationship among USP15 expression, YY1 expression, and HIV-1 gene expression and virus production, we also performed USP15 siRNA knockdown experiments. We first identified two USP15 siRNA to be effective in knocking down USP15 compared to the siRNA control (**Figures 11A–D**). Knockdown of USP15 using these two siRNA was associated with detection of decreased YY1 and increased of p24. In parallel, there were increases in HIV-1 transcription (both unspliced and total HIV-1 RNA transcripts, **Figures 11E, F**), and HIV-1 virus production (**Figure 11G**). There were clear trends of more HIV-1 unspliced RNA and total RNA in USP15 siRNA #2-transfected cells than the siRNA control-transfected cells. However, the differences did not achieve statistically significant, due to the high variations and standard deviations among the biological repeats. These results together provide additional evidence to support the notion that YY1 is directly involved in USP15-mediated inhibition of HIV-1 transcription, gene expression and virus production.

## 4 Discussion

Using the USP15 UbV inhibitors, we demonstrated that ectopic USP15 expression inhibited HIV-1 transcription, contributing to decreased HIV-1 gene expression and virus production, whereas expression of UbV, USP15 inhibitors, had opposite effects on HIV-1 transcription, gene expression and virus production. The transcription repression provides a new mechanism for USP15 inhibition of HIV-1 replication, preceding the previously reported USP15-targeted Nef and Gag degradation (Pyeon et al., 2016). The extent to which HIV-1 transcription changes from ectopic USP15 expression or UbV expression suggests the new mechanism to be primarily responsible for USP15-induced HIV-1 inhibition. The subsequent transcription factor activation array revealed YY1 and SRF as two potential transcription factors involved in the transcription inhibition of the HIV-1 LTR promoter. The more significant changes in YY1 than SRF in response to USP15 or UbV expression in the context of HIV-1, the known inhibitory function of YY1 in HIV-1 transcription, and the lack of any documented evidence between SRF activation and HIV-1 transcription inhibition prompted us to focus only on YY1 for the mechanistic studies.YY1 is a ubiquitously expressed and multi-functional transcription factor with four zinc-finger DNA-binding motifs in its C-terminal region (Hariharan et al., 1991; Park and Atchison, 1991; Gordon et al., 2006). YY1 inhibition of HIV-1 transcription has been well established through direct interaction with the LTR promoter or indirect engagement with other transcription factors. There are two putative YY1 DNA-binding sites within the HIV-1 LTR promoter (-16 to +27 nt. and -120 to -140 nt.) for indirect or direct binding of YY1 to the HIV-1 LTR promoter (Margolis et al., 1994; Malcolm et al., 2007; Bernhard et al., 2013). These sites are highly conserved among all HIV-1 isolates, including those three NL4-3, 89.6, and YU-2 used in this study. YY1 also interacts with late simian virus 40 factor (LSF) and recruitment of histone deacetylase 1 through its glycine/alanine-rich domain to the nuc1 region of the HIV-1 LTR promoter, resulting in nuc1 hypoacetylation and LTR repressive state (Margolis et al., 1994; Romerio et al., 1997; Coull et al., 2000; He and Margolis, 2002). The binding of LSF to the HIV-1 LTR promoter is a prerequisite for this process and that LSF engagement at LTR is regulated by the phosphorylation of LSF through mitogen-activated protein kinases (Ylisastigui et al., 2005). An instant suppression in HIV-1 LTR activation by ectopic YY1 expression and detection of a higher expression level of endogenous YY1 in HIV-1 latent cells, ACH-2, prior to reactivation support the notion that YY1 may be involved in the establishment or maintenance of HIV latency following the infection (Krishnan and Zeichner, 2004; Bernhard et al., 2013). YY1 may also play a role in HIV-1 infectivity and pathogenesis through down-regulation of the expression of CXCR4 and CCR5, two major HIV-1 co-receptors (Bleul et al., 1996; Choe et al., 1996; Dragic et al., 1996; Oberlin et al., 1996; Moriuchi et al., 1999; Moriuchi and Moriuchi, 2003). It is interesting to note in one study that ectopic YY1 expression increases HIV-1 transcription and viral production (Lee Yu et al., 2020), which was attributed to a different transfection efficiency control and a different sampling time of virus production.

Consistent with the roles of USP15 in the ubiquitin-proteasome pathway, we showed that there were no changes in YY1 mRNA expression, but there were significant increases in YY1 protein expression and the trend of increase in deubiquitination by ectopic USP15 expression or significant decreases of YY1 protein expression and the trend of decrease in deubiquitination by UbV expression. We further showed that USP15 and YY1 were detected in the same complex. In fact, YY1 has been shown to partake in distinct interplays and physical/functional interactions with several host cell and/or viral factors/promoters, modulating the transcription of various viruses (Seto et al., 1991; Shi et al., 1991; Lee et al., 1993; Seto et al., 1993; Shrivastava et al., 1993; Liu et al., 1994; Usheva and Shenk, 1994; Chiang and Roeder, 1995; Lee et al., 1995; Lewis et al., 1995; Montalvo et al., 1995; Bauknecht et al., 1996; O’Connor et al., 1996; Yang et al., 1996; Austen et al., 1997; Huang et al., 2008; Khalil et al., 2014; Shang et al., 2015; Lee et al., 2018; Warowicka et al., 2022). Our findings in the current study reveal that YY1 is a new cellular target for USP15-mediated deubiquitination and protein stabilization. Besides HIV-1 Nef and Gag (Pyeon et al., 2016), USP15 has been shown as a stabilizer of various cellular proteins/factors in the cytoplasm and nucleus and a regulator of multiple processes/pathways via its deubiquitinating activity. Examples are E3 ubiquitin ligase MDM2, regulating T cell activation (Zou et al., 2014); HIV-1 Tat-interacting protein of 110 kDa, regulating HIV-1 replication (Timani et al., 2014); Kelch-like ECH-associated protein 1 (Keap1), suppressing NF-E2-related factor 2 (Nrf2)-keap1 pathway (Villeneuve et al., 2013); E3 ligase SMURF2, enhancing the stability of TGF-β receptor (Iyengar et al., 2015); receptor-activated SMADs or TGF-β receptor I, modulating TGF-β signaling (Inui et al., 2011; Eichhorn et al., 2012); and transcriptional factor NF-E2-related factor 1 (Nrf1), maintaining protein homeostasis within the cell (Fukagai et al., 2016). The USP15 catalytic activity has also been linked to Parkin-mediated mitophagy, as its overexpression or knockdown leads to decreased or increased mitophagy, respectively (Cornelissen et al., 2014; Jacomin et al., 2018). In the current study, we demonstrated that YY1 served as a new target of the USP15 deubiquitinase activity. In addition, YY1 binds USP21, another USP family, and becomes deubiquitinated by USP21 with no changes in its transcription (Xu et al., 2020). Furthermore, YY1 also directly interacts with retroviral integrases, including HIV-1 integrase, and enhances viral DNA integration into host chromosomes (Inayoshi et al., 2010). These findings together suggest that post-translational modifications such as ubiquitination/deubiquitination may be a major regulatory mechanism of YY1 transcriptional function and that YY1 may be involved in other steps of HIV-1 replication.

In the study, we were only able to see a noticeable reduction in the level of YY1 protein in nuclear lysates of UbV transfected cells compared to both ectopic USP15-expressing cells and control cells without exogenous USP15 or UbV, although we could detect a higher level of YY1 protein in the whole cell lysate and cell nuclear fraction in cells transfected with USP15. The nuclear localization and presence of endogenous YY1 in or association with the nuclear matrix could be linked to a possible nuclear localization signal embraced within the second and third zinc fingers of the YY1 encoding region (Austen et al., 1997), the C-terminal domain (amino acid 257-341) of YY1 (McNeil et al., 1998), or through the formation of a complex between YY1 and shuttling nucleolar proteins like B23 (Borer et al., 1989; Inouye and Seto, 1994). Several mechanisms have been shown to regulate the specificity and selectivity of DUB for substrates, including changes of DUB subcellular distribution (Clague et al., 2012). USP15 has been proposed to possess a nuclear localization signal and detected in the cytoplasm, nucleus, and nucleolus, depending upon cell type and post-translational modifications (Soboleva et al., 2005; de Jong et al., 2006; Das et al., 2019). As such, in this study, we could detect exogenous USP15 in both cytoplasmic and nuclear compartments.

There are a couple of caveats about the current study. Two UbV inhibitors, UbV15.1 and UbV15.1/D, were used in the experiments. The former is more selective to USP15 than USP4, USP11, and USP19, while the latter is highly specific to USP15 (Teyra et al., 2019). USP15 shares significant structural and sequence similarity with USP4 and USP11 (Vlasschaert et al., 2015; Das et al., 2017; Teyra et al., 2019). Thus, it is likely that USP4 and USP11 would have a similar function on YY1 stability and inhibit HIV-1 transcription. The second caveat is the effects of UbV expression on the cell cycle in the presence of HIV-1. We noticed fewer cells in the transfection with HIV-1 and UbV plasmids than that with HIV-1 only. Additional cell cycle analyses showed that there were more cells at G2/M in the transfections with both HIV-1 and UbV than in the transfections with HIV-1 only, supporting the roles of USP15 in the cell cycle (Faronato et al., 2013; Ren et al., 2023). The relationship between the USP15 regulation of cell cycle and its inhibition of HIV-1 transcription merits further investigation.

In summary, YY1 directly binds to the putative DNA-binding sites within the HIV-1 LTR promoter or interacts with other transcription factors that have DNA-binding sites within the HIV-1 LTR promoter and inhibits HIV-1 gene transcription. The homeostasis of YY1 is maintained by the balance between deubiquitinase USP15 and E3 ubiquitin ligase. Ectopic USP15 expression deubiquitinates and stabilizes endogenous YY1 and thus suppresses HIV-1 transcription, while USP15-specific UbV inhibitors prevent endogenous USP15 from deubiquitinating YY1, thus promoting YY1 degradation and relieving USP15-induced inhibition of HIV-1 transcription.

## Data Availability

The original contributions presented in the study are included in the article/**Supplementary Materials**, further inquiries can be directed to the corresponding author/s.

## Supplementary material

Supplementary Figure 1 Effects of ectopic UbV expression on HIV-1 gene expression by immunofluorescence staining. 293T were cultured on pre-coated (with poly-L-lysine) glass coverslips, transfected with pNL4-3 or pNL4-3 plus pFlag-UbV15.1 (1:1 ratio). The transfection efficiency was close to 100%. The Cells were fixed for immunofluorescence staining against an anti-p24 antibody and then a goat anti-mouse IgG (H+L) secondary antibody, Alexa Fluor™ 555, and DAPI nuclei counterstaining. Representative images were taken using a TRITC filter (red for p24) and a DAPI filter (blue for nuclei) with 20x magnification **(A)**. The ImageJ software was used to calculate the relative mean fluorescence intensity (MFI) with normalization to cell counts determined by DAPI staining; the data were Mean ± SD of four replicates (n = 4, **B**).

Supplementary Figure 2 Relationship between ectopic UbV expression and HIV-1 gene expression of HIV-1 89.6 and YU-2. 293T were transfected with p89.6 **(A)**, pYU-2 **(B)**, or pFlag-UbV15.1 or pFlag-UbV15.1/D or HIV-1 isolates plus pFlag-UbV15.1 or pFlag-UbV15.1/D (0.5,1.0, and 2.0 µg). pcDNA3 was included to equalize the total DNA amounts among the transfections. The cells were harvested for whole cell lysates, followed by Western blotting against anti-p24, anti-Flag, anti-GAPDH, or anti-β-actin antibody.
