# Supplementary material for: Ectopic USP15 expression inhibits HIV-1 transcription involving changes in YY1 deubiquitination and stability
Source: Front Cell Infect Microbiol. 2024 Nov 18;14:1371655. doi: 10.3389/fcimb.2024.1371655 (PMC11609158; doi:10.3389/fcimb.2024.1371655)
Supplement: Supplementary Figure 1 — Effects of ectopic UbV expression on HIV-1 gene expression by immunofluorescence staining. 293T were cultured on pre-coated (with poly-L-lysine) glass coverslips, transfected with pNL4-3 or pNL4-3 plus pFlag-UbV15.1 (1:1 ratio). The transfection efficiency was close to 100%. The Cells were fixed for immunofluorescence staining against an anti-p24 antibody and then a goat anti-mouse IgG (H+L) secondary antibody, Alexa Fluor™ 555, and DAPI nuclei counterstaining. Representative images were taken using a TRITC filter (red for p24) and a DAPI filter (blue for nuclei) with 20x magnification (A). The ImageJ software was used to calculate the relative mean fluorescence intensity (MFI) with normalization to cell counts determined by DAPI staining; the data were Mean ± SD of four replicates (n = 4, B). [file DataSheet1.docx]

Supplementary Material

# Supplementary Figures

## Supplementary Figures


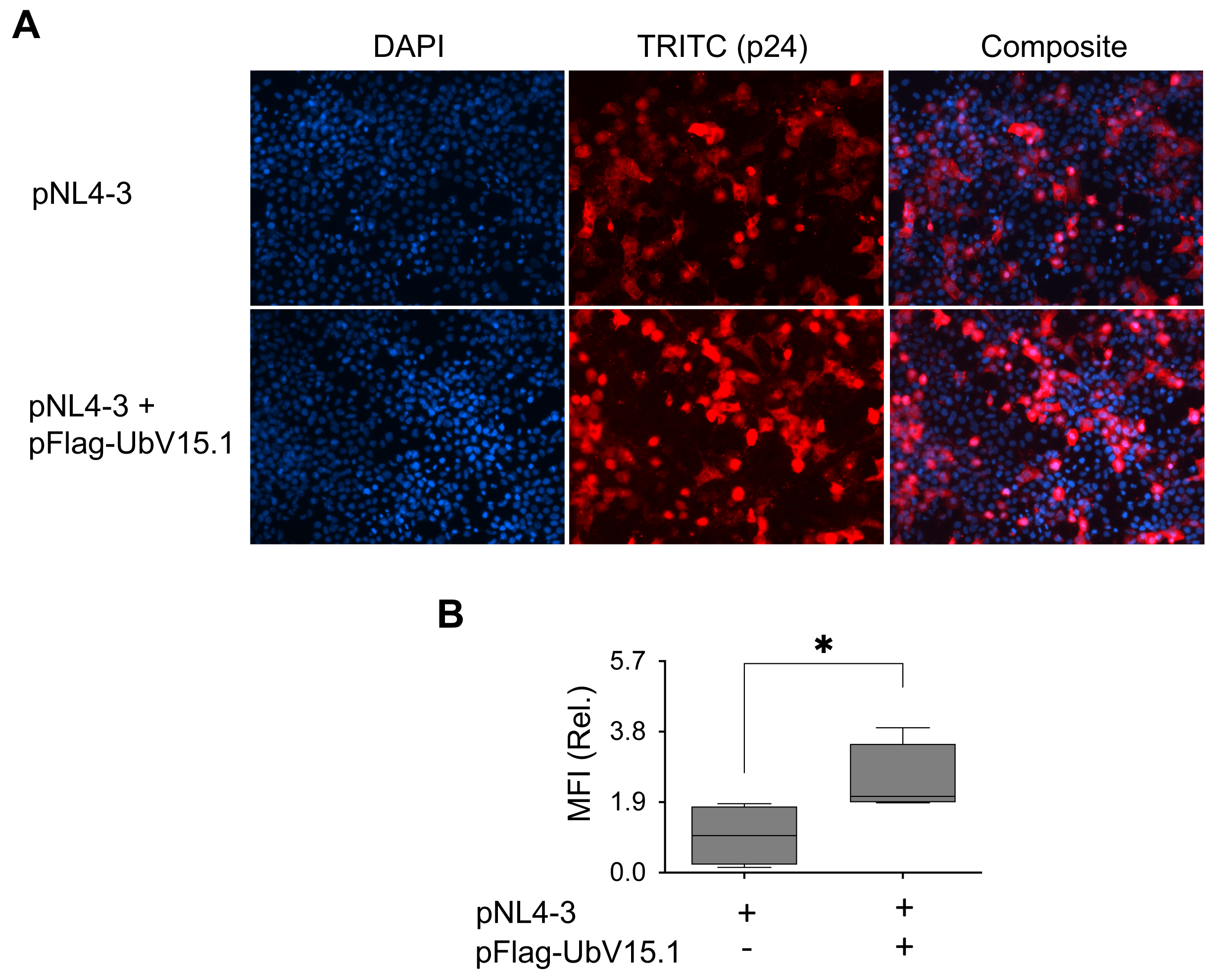


**Supplementary Figure 1.** **Effects of ectopic UbV expression on HIV-1 gene expression by immunofluorescence staining.** 293T were cultured on pre-coated (with poly-L-lysine) glass coverslips, transfected with pNL4-3 or pNL4-3 plus pFlag-UbV15.1 (1:1 ratio). The transfection efficiency was close to 100%. The Cells were fixed for immunofluorescence staining against an anti-p24 antibody and then a goat anti-mouse IgG (H+L) secondary antibody, Alexa Fluor™ 555, and DAPI nuclei counterstaining. Representative images were taken using a TRITC filter (red for p24) and a DAPI filter (blue for nuclei) with 20x magnification (**A**). The ImageJ software was used to calculate the relative mean fluorescence intensity (MFI) with normalization to cell counts determined by DAPI staining; the data were Mean ± SD of four replicates (n = 4, **B**)


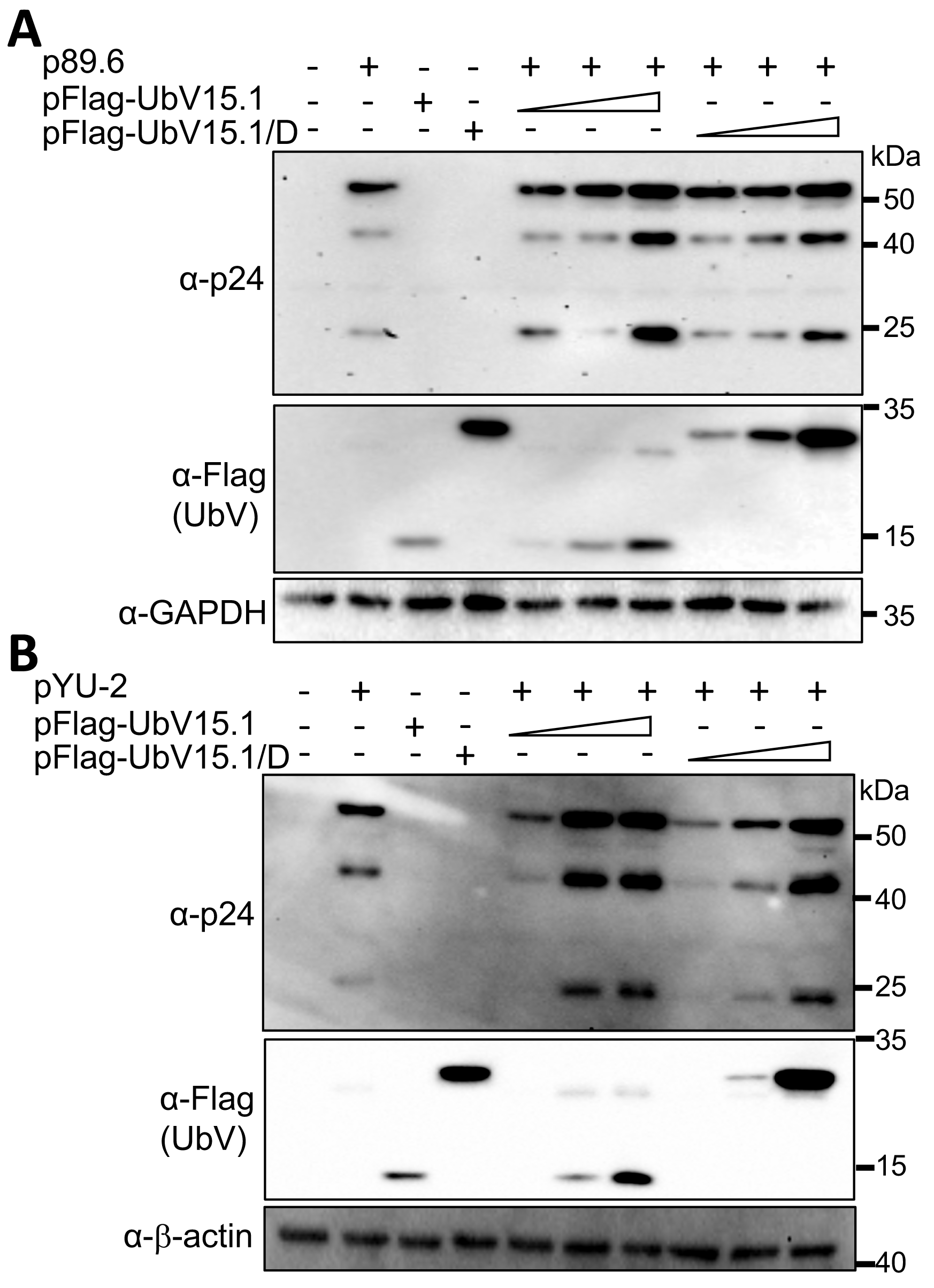


**Supplementary Figure 2. Relationship between ectopic UbV expression and HIV-1 gene expression of HIV-1 89.6 and YU-2.** **A.** 293T were transfected with p89.6 (**A**), pYU-2 (**B**), or pFlag-UbV15.1 or pFlag-UbV15.1/D or HIV-1 isolates plus pFlag-UbV15.1 or pFlag-UbV15.1/D (0.5,1.0, and 2.0 µg). pcDNA3 was included to equalize the total DNA amounts among the transfections. The cells were harvested for whole cell lysates, followed by Western blotting against anti-p24, anti-Flag, anti-GAPDH, or anti-β-actin antibody.
